# Functional Analysis of Amino Acid Residues Responsible for Substrate Specificity of GH13_17 α‑Glucosidase from *Aedes aegypti* Saliva (AaMalI)

**DOI:** 10.1021/acsomega.5c08180

**Published:** 2026-03-02

**Authors:** Waraporn Auiewiriyanukul, Wataru Saburi, Haruhide Mori, Dumrongkiet Arthan, Sorachat Tharamak

**Affiliations:** † Department of Biochemistry, Faculty of Science, 426949 Kasetsart University, 50 Ngamwongwan, Chatuchak, Bangkok 10900, Thailand; ‡ Research Faculty of Agriculture, 12810 Hokkaido University, Kita 9 Nishi 9, Sapporo 060-8589, Japan; § Department of Tropical Nutrition and Food Science, Faculty of Tropical Medicine, 683980 Mahidol University, Bangkok 10400, Thailand; ∥ Department of Chemistry and Center of Excellence for Innovation in Chemistry, Special Research Unit for Advanced Magnetic Resonance, Faculty of Science, Kasetsart University, 50 Ngamwongwan, Chatuchak, Bangkok 10900, Thailand

## Abstract

The α-glucosidase (AaMalI) in *Aedes aegypti* saliva belongs to glycoside hydrolase family 13, subfamily 17 (GH13_17) and plays a crucial role in the digestion of sucrose, which is the main sugar involved in insect metabolism. The amino-acid residues in the conserved region II have been reported as the key residues for sucrose specificity in GH13_17. Using mutagenesis, this study expressed and purified recombinant AaMalI and determined the molecular mechanism related to substrate specificity. The optimal activity was at pH 6.3 and 40 °C. AaMalI had a trisaccharide specificity similar to GH13 α-glucosidases and preference for sucrose over maltose. The single mutation of Y223H and the double mutation of P222N/Y223H altered the substrate preference from sucrose to maltose. Structural analysis of the AaMalI model obtained by superimposition with the maltose-bound complex suggested that Tyr292 stabilizes the d-glucosyl moiety at subsite +1, whereas His223 indirectly contributes to maltose hydrolysis. These findings provide structural insights into AaMalI substrate specificity and support its potential as a target for vector mosquito control.

## Graphical abstract

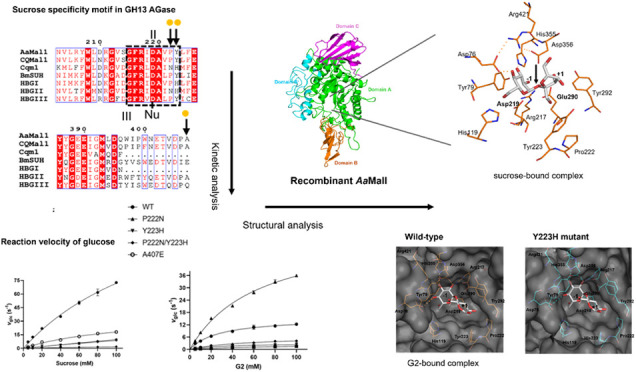

## Introduction

α-Glucosidase (EC 3.2.1.20, AGase) is a retaining glycosidase that hydrolyzes α-glucosidic linkage at the nonreducing end of substrates.^1^ This enzyme is found in various organisms, including bacteria, yeast, fungi, archaea, plants, and animals.^2–7^ AGase, which belongs to glycoside hydrolase family 13 (GH13) in the sequence-based classification of glycoside hydrolases,^8^ is similar structurally to various amylolytic enzymes such as α-amylase (EC 3.2.1.1), oligo-1,6-glucosidase (EC 3.2.1.10), pullulanase (EC 3.2.1.41), isoamylase (EC 3.2.1.68), amylosucrase (EC 2.4.1.4), and sucrose α-1,2-glucosidase (EC 3.2.1.48). According to the subfamily classification of GH13 enzymes based on phylogenetic analysis,^9,10^ AGase falls in subfamilies 17, 23, 30, 31, 40, and 44. GH13 AGase has three domains, A–C, which are commonly found in the other GH13 enzymes. Domain A is the catalytic domain, formed by a (β/α)_8_ barrel-structure; domain B is a long loop between the third β-strand and the third α-helix of domain A; domain C, as with domain A, is formed by antiparallel β-sheets. The catalytic nucleophile (Asp) and general acid/base (Glu) are located at the C-terminal of the fourth and fifth β-strands of domain A, respectively. A protruding β→α loop 8 in domain A contains two α-helices and is referred to as domain B’. This domain interacts with domain B and contributes to the formation of a pocket-shaped active site.^11,12^ The four classical conserved sequence regions of GH13 enzymes are located at the C-terminal ends of the third, fourth, fifth, and seventh β-strands of domain A, respectively, with the residues included in the conserved regions being involved in the formation of subsite −1.^13^ AGase is divided into three groups depending on the substrate specificity:^14^ Group I prefers sucrose and aryl α-glucosides, whereas groups II and III prefer homogeneous substrates such as maltose and longer maltooligosaccharides. Group III has high activity toward long-chain substrates (starch and glycogen).

AGase from insects is classified into GH13 subfamily 17 (GH13_17). This classification is relevant since different GH13 subfamilies display distinct structural and functional features, providing an evolutionary context to understand the substrate specificity of insect AGase. During development and reproduction, insect AGase is involved in converting nectar-derived carbohydrate into simple sugars that support survival, flight, reproduction, and overall metabolism.^15,16^ Other studies of mosquitoes (*Aedes aegypti*, *Aedes albopictus*, *Anopheles darlingi*, *Anopheles dirus*, and *Culex quinquefasciatus*) have reported the expression of AGase in the saliva.^17–19^ The AGase found in the salivary glands of mosquitoes plays a major role in sugar processing before blood feeding.^20^ The enzyme breaks down sucrose, which is the major component in nectar, and digests maltose (G2) and other glycosylated carbohydrates into glucose for ATP production. In addition to insects, AGase has also been characterized in fungi and yeasts, which employ a similar enzyme for carbohydrate metabolism. For instance, nectar-dwelling yeast plays a key ecological role in the tripartite interaction among angiosperms and pollinating insects, influencing nectar chemistry, insect behavior, and plant pollination.^21,22^ Many fungal and bacterial AGase share catalytic and mechanistic features with insect AGases toward efficient sugar utilization and transglycosylation at high substrate concentrations. A yeast maltase from *Blastobotrys adeninivoran*, BaAG2, a member of GH13, has been reported to exhibit broad substrate specificity, including hydrolytic activity on starch-like polymers and the ability to produce oligosaccharides through transglycosylation.^23^ This reaction is characteristic of AGase in the GH13 and GH31 families and proceeds via a double-displacement mechanism involving a covalent glycosyl-enzyme intermediate. Under high substrate concentrations, the enzyme favors transglycosylation over hydrolysis, using a sugar molecule as the acceptor.^24,25^ These studies highlight that AGase across diverse organisms shares conserved features despite their adaptation to different ecological niches.

Mosquito salivary AGase in *Ae. aegypti*, *An. gambiae*, and *C. quinquefasciatus* has been expressed successfully using a mammalian expression system and characterized.^26^ These recombinant enzymes displayed a preference for sucrose over G2, resembling the substrate specificity of the European honeybee (HBG) isozyme III.^27^ In European honeybees, three AGase isozymes (HBG-I, HBG-II, and HBG-III) have been reported in different organs and at different life stages. HBG-I was found in the midgut, HBG-II in the midgut and hemolymph, and HBG-III in the hypopharyngeal gland (a type of salivary gland) of adult honeybees.^28^ Previous studies on HBG-III demonstrated that Pro226 and Tyr227 (the third and fourth residues from the catalytic nucleophile toward C-terminus in the conserved region II) play a critical role in determining sucrose specificity.^29^ In AaMalI, a salivary gland AGase expressed in the *Komagataella phaffii* X-33 transformant, the corresponding residues are Pro222 and Tyr223, respectively. These residues are considered candidates for investigating whether similar mechanisms govern substrate recognition in mosquito AGase. Additionally, Ala407 of AaMalI, located at domain B’ that contributes to forming the substrate-binding cleft, corresponds to a Glu residue in GH13_17 family from Lepidoterans, which has been associated with high sucrase activity.^30^ Based on this insight, the present study aimed to investigate the enzyme function of AaMalI and carried out mutational analysis to elucidate the molecular mechanism underlying its substrate specificity at these critical residues.

## Materials and Methods

### Construction of AaMalI Expression Plasmid

Total RNA was extracted from *Ae. aegypti* using TRIzol reagent (Thermo Fisher Scientific; Waltham, MA). The single strand of cDNA was synthesized by using reverse transcriptase (Thermo Fisher Scientific; Waltham, MA) with the total RNA extract as a template. The cDNA encoding AaMalI was amplified using polymerase chain reaction (PCR) with KOD Hot Start DNA polymerase (Toyobo; Osaka, Japan). The primers were designed based on the sequences of *Ae. aegypti* maltase-like I gene (GenBank accession number: M30442.1). The amplified fragment, corresponding to the mature protein without signal peptide fused to a C-terminal (His)_6_-tag, was cloned into the pPICZαB vector (Invitrogen; Waltham, MA) at the EcoRI and NotI restriction sites via the pGEM-T/AaMalI intermediate construct. All primers used were listed in Table S1. The final construct, designated as pPICZαB/AaMalIHis, was confirmed using DNA sequencing by Macrogen (Seoul, Republic of Korea).

### Preparation of AaMalI Mutants

Expression plasmids of four mutants (P222N, Y223H, P222N/Y223H, and A407E) were constructed using a Primestar Mutagenesis Basal Kit (Takara Bio; Kusatsu, Japan). All of the primers used for mutant construction were listed in Table S1. The DNA sequences were confirmed using Applied Biosystems 3130 Genetic Analyzer software (Life Technologies; Carlsbad, CA). Predicted N-glycosylation sites were analyzed using the NetNGlyc server (Technical University of Denmark, Lyngby, Denmark),^31^ which confirmed that Pro222, Y223, and Ala407 were not located within any predicted glycosylation sites.

### Preparation of Recombinant AaMalI and Mutants

A sample of the expressed plasmid (10 μg) was linearized using SacI digestion and introduced into *K. phaffii* (Invitrogen, Waltham, MA) based on electroporation using the Gene Pulser Xcell electroporation system (Biorad; Hercules, CA). Transformants were screened on a yeast peptone dextrose sorbitol agar medium containing 20 g/L agar (Nacalai Tesque; Kyoto, Japan), 10 g/L yeast extract (Nacalai Tesque; Kyoto, Japan), 20 g/L peptone (Becton, Dickinson and Company; Franklin Lakes, NJ), 20 g/L d-glucose, 1 M sorbitol, and 100 μg/mL of zeocin. A positive transformant was grown in 1 L of buffered glycerol-complex (BMGY) medium (containing 10 g/L yeast extract, 20 g/L peptone, 0.1 M potassium phosphate buffer, 13.4 g/L yeast nitrogen base (Thermo Fisher Scientific; Waltham, MA), 0.4 mg/L biotin, and 10 g/L glycerol) at 30 °C with shaking until the absorbance at 600 nm reached 5 (20 h). The cells were harvested using centrifugation (7000*g*, 4 °C, 10 min) and resuspended in 250 mL of modified BMGY medium in which glycerol was replaced with methanol. The induction culture was performed at 16 °C for 5 days, with methanol supplemented at a final concentration of 5 g/L every 24 h to maintain the induction. The protein was precipitated based on overnight incubation of the cell-free culture supernatant at 4 °C in the presence of 80% saturated ammonium sulfate. Then, the precipitate was collected using centrifugation (7000 × *g*, 4 °C, 10 min), dissolved in 20 mM sodium phosphate buffer (pH 8.0), and dialyzed against the same buffer. Recombinant AaMalI was purified using a Ni-immobilized chelating Sepharose Fast Flow column (1.5 cm i.d. × 6 cm length; Cytiva; Uppsala, Sweden) that was equilibrated with 20 mM sodium phosphate buffer (pH 8.0) containing 0.3 M sodium chloride (buffer A). The nonabsorbed protein was eluted with buffer A, and the absorbed protein was eluted based on a linear gradient (0–0.3 M) of imidazole in the same buffer. The fractions with AGase activity were pooled and dialyzed against 20 mM sodium phosphate buffer (pH 6.0); then, they were subjected to gel permeation chromatography on a Toyopearl HW-55S column (2.6 cm i.d. × 100 cm length; Tosoh; Tokyo, Japan) that was equilibrated with 20 mM sodium phosphate buffer (pH 6.0). The active fractions collected were concentrated using ultrafiltration (Vivaspin 20; Sartorius; Göttingen, Germany). Mutant enzymes were produced and purified in the same fashion as that of the wild type. The protein purity of the purified samples was evaluated using sodium dodecyl sulfate–polyacrylamide gel electrophoresis (SDS–PAGE).^32^

### Deglycosylation of Recombinant Proteins

Purified wild-type and mutants of AaMalI (0.22 μg each) were heated at 100 °C for 3 min in 105 μL of 20 mM sodium acetate buffer (pH 5.0). Endoglycosidase H (25 mU; 5 U/mL; Roche Diagnostics; Indianapolis, IN) was then added, and the mixture was incubated at 37 °C for 16–18 h. The resulting products were analyzed by using SDS–PAGE, and protein bands were visualized with Rapid CBB Kanto (Kanto Chemical; Tokyo, Japan).

### Determination of Protein Concentration

The protein concentration of the cell-free supernatant was determined using the Bradford assay,^33^ in which bovine serum albumin (BSA) was used as a standard. During column chromatography, protein concentrations were monitored by UV absorbance at 280 nm, assuming an extinction coefficient of *E*_0.1%_ = 1.0 (equivalent to 1 mg/mL protein). In addition, the concentrations of purified samples were determined by the Institute of Analysis Division (Global Facility Center, Hokkaido University, Japan) based on amino-acid analysis of complete protein hydrolysates in 6 M HCl, which allowed the quantification of the theoretical amino-acid contents.

### Standard Enzyme Activity Assay

A sample of the reaction mixture (100 μL), containing the glycosylated enzyme and 2 mM *p*-nitrophenyl α-glucopyranoside (pNPGlc; Fujifilm Wako Pure Chemical; Osaka, Japan) in 50 mM sodium phosphate buffer (pH 6.0), was incubated at 37 °C for 10 min. The reaction was terminated by adding 200 μL of 1 M Na_2_CO_3_. Absorbance at 405 nm was measured; the liberated *p*-nitrophenol (pNP) concentration was determined from a standard curve of pNP (0–0.16 mM), where one unit of enzyme activity was defined as the amount of the enzyme that hydrolyzed 1 μmol of pNPGlc in 1 min under these conditions.

### Effect of pH and Temperature on Activity and Stability

The optimal pH and temperature were investigated based on the enzyme activity at various pH values and temperatures, respectively. The reaction pH was changed using 80 mM Britton–Robinson buffer (pH value of the mixture of acetate, phosphate, and glycine was adjusted using 6 M NaOH; pH 3.0–11.5). pH stability was assessed by measuring the residual activity after incubation of the enzyme at the initial concentration of 0.37 U/mL in 80 mM Britton–Robinson buffer (pH 3.0–11.5) at 37 °C for 20 min. Thermal stability was evaluated by incubating the enzyme at an initial concentration of 0.37 U/mL in 50 mM sodium phosphate buffer (pH 6.0) at temperatures ranging from 6 to 60 °C for 20 min. After incubation, the residual enzyme activity was determined using final enzyme concentrations of 4.91 and 7.37 mU/mL for pH and temperature assays, respectively.

### Kinetic Analysis of Reactions with Various Substrates

The reaction rates of various substrates were measured under the conditions of the standard enzyme assay: maltose (G2; Nihon Shokuhin Kako; Tokyo, Japan), maltotriose (G3; Nihon Shokuhin Kako; Tokyo, Japan), maltotetraose (G4; Nihon Shokuhin Kako; Tokyo, Japan), sucrose (Fujifilm Wako Pure Chemical; Osaka, Japan), isomaltose (Tokyo Chemical Industry; Tokyo, Japan), trehalose (Hayashibara; Okayama, Japan), nigerose (Hayashibara; Okayama, Japan), kojibiose (Fujifilm Wako Pure Chemical; Osaka, Japan), and pNPGlc. The substrate concentration ranges were 5–100 mM for sucrose and G2, 0.3–15 mM for G3 and G4, and 0.5–12 mM for pNPGlc. Each 100 μL of reaction mixture contained enzyme at final concentrations ranging from 32.7 nM to 2.87 μM, depending on the variant tested. The reactions with the substrates other than pNPGlc were stopped by adding 50 μL of 4 M Tris-HCl buffer (pH 7.0), and the liberated d-glucose was determined using a Glucose C-II Test Kit (Fujifilm Wako Pure Chemical; Osaka, Japan). The standard was 0–250 μM d-glucose (Nacalai Tesque; Kyoto, Japan). Nonlinear regression fitting of reaction velocities to the Michaelis–Menten equation was performed using Grafit version 7.0.2 software (Erithacus Software; East Grinstead, U.K.).

### TLC Analysis of Transglucosylation Products

AaMalI (0.7 μM) was incubated with 0.5 M of each of G2, G3, and sucrose in 50 mM sodium phosphate buffer (pH 6.0) at 37 °C for 3 h. The reaction was stopped by heating the sample to 100 °C for 5 min. The G2 and G3 samples were developed using a solvent containing 2-propanol/1-butanol/water = 12/3/4 (v/v/v). A developing solvent containing chloroform/acetic acid/water = 6:6:1 (v/v/v) was used for the sample containing sucrose. The carbohydrates were visualized based on heating after spraying a detection reagent of anisaldehyde/sulfuric acid/acetic acid = 1/2/100 (v/v/v).

For the analysis of transglucosylation products from pNPGlc, AaMalI (0.7 μM) was reacted with 10 mM pNPGlc. After the reaction had been stopped by heating, the sample was analyzed to identify the reaction products from sucrose, G2 and G3 under the same conditions.

### Protein Structure Prediction and Structural Comparison

The amino-acid sequences of wild-type AaMalI and its mutants were obtained from experimentally determined sequences, and all sequences were prepared in FASTA format. The three-dimensional structures of all variants were predicted using AlphaFold3.^34^ Models with high pLDDT confidence scores were selected for subsequent analyses. To investigate the structural features related to substrate binding, crystal structures of GH13 AGase in complexes with different ligands were retrieved from the Protein Data Bank (PDB; rcsb.org). These included the *Bombyx mori* sucrose hydrolase (BmSUH) E322Q mutant bound to sucrose (PDB entry, 6LGG)^30^ and the *Bacillus* sp. AHU2216 AGase (BspAG13_31A) E256Q mutant bound to G2, G3, and G4 (PDB entries, 5ZCC, 5ZCD, and 5ZCE, respectively).^12^ The coordinates of sucrose, G2, G3, and G4 were directly derived from these PDB structures and used as reference ligand coordinates for structural superimposition, comparison, and visualization in the AaMalI models using PyMOL.^35^ The alignments were based on Cα atoms to minimize the overall structural deviation. RMSD values were calculated to evaluate the structural similarity between the predicted and reference structures. Ligand orientation and substrate binding within the catalytic pocket were examined. Structural differences between the wild-type and mutant proteins were analyzed by examining the residue orientation, side-chain positioning, and pocket geometry. All structural visualizations and figures were generated by using PyMOL.

## Results

### Characterization of Recombinant Wild-Type AaMalI

Recombinant AaMalI was produced in *K. phaffii*, and 1.8 mg of purified enzyme was obtained from 250 mL of the culture broth (Table 1). The enzyme had pNPGlc-degrading activity (1.32 U/mg). A smear protein band of 80 kDa was detected in the SDS–PAGE analysis. After deglycosylation with endoglycosidase H, a predominant band of 67 kDa was observed, which corresponds to the molecular weight calculated from the amino-acid sequence. This suggests that recombinant AaMalI was N-glycosylated through production in *K. phaffi* (Figure S1). The enzyme had optimal activity at pH 6.0 and 40 °C. It retained over 90% of maximal activity within the temperature range of 26–37 °C and pH range of 6.5–8.2 (Figure 1).

**Figure 1:**
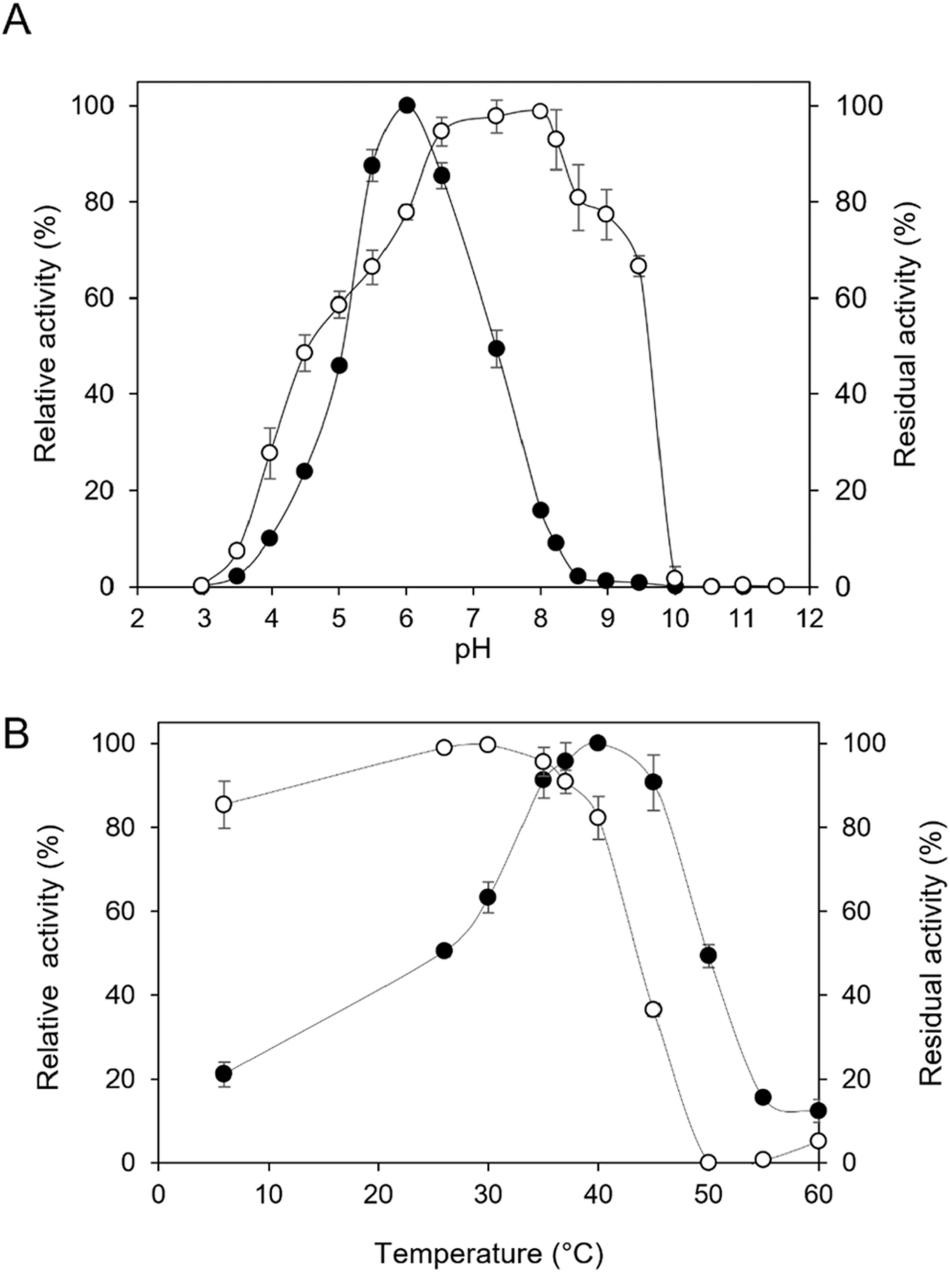
Effects of pH and temperature on enzyme activity (●) and stability (○) of AaMalI. (A) Effect of pH on enzyme activity and stability. AaMalI activity was measured at different pH values using 80 mM Britton–Robinson buffer (pH 3.0–11.5). The stability of the enzyme at different pH levels was assessed based on measuring the residual activity after pH treatment at 37 °C for 20 min. (B) Effect of temperature on enzyme activity and stability. AaMalI activity was measured at various temperatures (6–60 °C). Thermal stability was determined by incubating the enzyme in 50 mM sodium phosphate buffer (pH 6.0) at temperatures ranging from 6 to 60 °C for 20 min, followed by the determination of the residual activity. The activity was measured using the pNP method.

**Table 1:** Summary of Purification Procedures of Recombinant AaMalI

procedure	protein (mg)	activity (U)	specific activity^a^ (U mg^–1^)	yield (%)	purification (-fold)
cell-free supernatant^b^	339	17.3	0.05	100	1.00
80% sat. ammonium sulfate^b^	274	17.2	0.06	99.4	1.23
nickel-sepharose chromatography^c^	30.1	9.20	0.14	53.2	2.90
gel-filtration^c^	11.1	5.48	0.49	31.7	9.67
ultracentrifugation^d^	1.80	2.37	1.32	13.7	25.8

a Specific activity calculated from enzyme activity divided by the amount of protein using pNPGlc as a substrate.

b Amount of protein measured using the Bradford method.

c Amount of protein determined using the UV method (*A*_280_).

d Amount of protein of purified sample after ultracentrifugation calculated based on protein extinction coefficient (*E*_0.1%_).

Reaction rates toward various AGase were measured, with AaMalI having high velocity to sucrose and maltooligosaccharides as natural substrates but with hardly any hydrolyzed glucobioses other than G2 (Table 2). The reactions on the predominant substrates followed Michaelis–Menten kinetics, and the parameters were determined from the saturation curves (Table 3 and Figure S2). AaMalI had substantially high catalytic efficiency (*k*_cat_/*K*_m_) values for G3 (23.5 s^–1^ mm ^–1^) and G4 (11.4 s^–1^ mm ^–1^), which were 112- and 54.3-fold higher than that for G2. In addition, the *k*_cat_/*K*_m_ for sucrose (1.14 s^–1^ mm ^–1^) was 5.43-fold higher than that for G2 (0.210 s^–1^ mm ^–1^). Notably, the *k*_cat_ and *K*_m_ values were characteristically high for sucrose, as observed in HBG-III.

**Table 2:** Reaction Rate of Wild-Type to Various Substrates^a^

substrate		rate (s^–1^)
G2	α-d-Glc*p*-(1→4)-α-d-Glc*p*	0.39 ± 0.01
G3	α-d-Glc*p*-(1→4)-α-d-Glc*p*-(1→4)-α-d-Glc*p*	17.1 ± 0.15
G4	α-d-Glc*p*-(1→4)-α-d-Glc*p*-(1→4)-α-d-Glcp-(1→4)-α-d-Glc*p*	6.97 ± 0.16
sucrose	α-d-Glc*p*-(1→2)-β-d-Fruc*f*	2.15 ± 0.03
isomaltose	α-d-Glc*p*-(1→6)-α-d-Glc*p*	(0.82 ± 0.47) × 10^–3^
nigerose	α-d-Glc*p*-(1→3)-α-d-Glc*p*	(2.50 ± 0.12) × 10^–2^
kojibiose	α-d-Glc*p*-(1→2)-α-d-Glc*p*	(1.82 ± 0.12) × 10^–2^
trehalose	α-d-Glc*p*-(1→1)-α-d-Glc*p*	(1.87 ± 0.35) × 10^–3^
pNPGlc	pNP-α-Glc*p*	1.41 ± 0.02

a Reaction rate for hydrolysis is shown using 2 mM substrate concentrations. G2, G3, G4, and pNPGlc represent maltose, maltotriose, maltotetraose, and *p*-nitrophenyl α-glucopyranoside, respectively. Values presented as average ± SD from three independent experiments.

**Table 3:** Kinetic Parameters of AaMalI Variants^a^

enzyme	parameter (unit)	sucrose	G2	G3	G4	pNPGlc
wild type	*k*_cat_ (s^–1^)	193 ± 6.00	5.86 ± 0.19	25.4 ± 1.00	20.8 ± 0.30	25.3 ± 1.60
	*K*_m_ (mM)	169 ± 14.0	27.9 ± 2.40	1.08 ± 0.08	1.83 ± 0.03	7.15 ± 0.96
	*k*_cat_/*K*_m_ (s^–1^ mM^–1^)	1.14 (543)	0.21 (100)	23.5 (11,200)	11.4 (5430)	3.54 (1690)
P222N	*k*_cat_ (s^–1^)	81.6 ± 11.8	2.67 ± 0.19	8.15 ± 1.57	4.59 ± 0.13	13.3 ± 0.50
	*K*_m_ (mM)	705 ± 111	75.8 ± 11.2	6.42 ± 0.87	7.87 ± 0.78	6.74 ± 0.50
	*k*_cat_/*K*_m_ (s^–1^ mM^–1^)	0.12 (330)	0.03 (100)	1.27 (3610)	0.58 (1660)	1.97 (5600)
Y223H	*k*_cat_ (s^–1^)	25.0 ± 2.80	54.4 ± 1.84	15.2 ± 0.10	17.9 ± 0.20	43.3 ± 2.00
	*K*_m_ (mM)	186 ± 29.0	49.4 ± 2.92	1.37 ± 0.09	2.89 ± 0.09	12.3 ± 1.10
	*k*_cat_/*K*_m_ (s^–1^ mM^–1^)	0.13 (12.2)	1.10 (100)	11.1 (1010)	6.19 (563)	3.52 (320)
P222N/Y223H	*k*_cat_ (s^–1^)	5.70 ± 0.75	3.09 ± 0.14	5.38 ± 0.05	5.18 ± 0.14	10.4 ± 0.30
	*K*_m_ (mM)	190 ± 33.0	30.6 ± 1.30	1.52 ± 0.02	3.21 ± 0.31	3.57 ± 0.09
	*k*_cat_/*K*_m_ (s^–1^ mM^–1^)	0.03 (29.7)	0.10 (100)	3.54 (3500)	1.61 (1590)	2.91 (2880)
A407E	*k*_cat_ (s^–1^)	33.1 ± 4.20	6.09 ± 0.57	15.9 ± 0.50	10.6 ± 0.40	3.68 ± 0.19
	*K*_m_ (mM)	79.5 ± 21.0	44.4 ± 7.43	2.48 ± 0.15	2.90 ± 0.07	11.1 ± 1.30
	*k*_cat_/*K*_m_ (s^–1^ mM^–1^)	0.42 (304)	0.14 (100)	6.41 (4680)	3.66 (2670)	0.33 (242)

a G2, G3, G4, and pNPGlc represent maltose, maltotriose, maltotetraose, and *p*-nitrophenyl α-glucopyranoside, respectively. Values presented as average ± SD for three independent experiments. Values in parentheses are the percentage to *k*_cat_/*K*_m_ for G2.

### Transglucosylation Products

Transglucosylation activity was determined by analyzing the reaction products of AaMalI from 0.5 M G2, G3, and sucrose on TLC. AaMalI produced longer oligosaccharides of G3 from G2 after 2 h (Figure 2A). From sucrose, erlose (α-d-Glc*p*-(1→4)-α-d-Glc*p*-(1↔2)-β-d-Fruc*f*) was generated after 1 h (Figure 2C). In the case of G3 as a substrate, the formation of G4 was already detectable within 5 min (Figure 2B). The identities of G3, G4, and erlose were identified based on comparison with authentic sugar standards on TLC. With pNPGlc as a substrate, the enzyme produced a putative transglucosylation product that migrated slowly than pNPGlc (Figure 2D), which was presumed to correspond to a pNP α-maltoside. These results indicate that AaMalI catalyzed the formation of an α-(1→4)-glucosidic linkage through transglucosylation.

**Figure 2:**
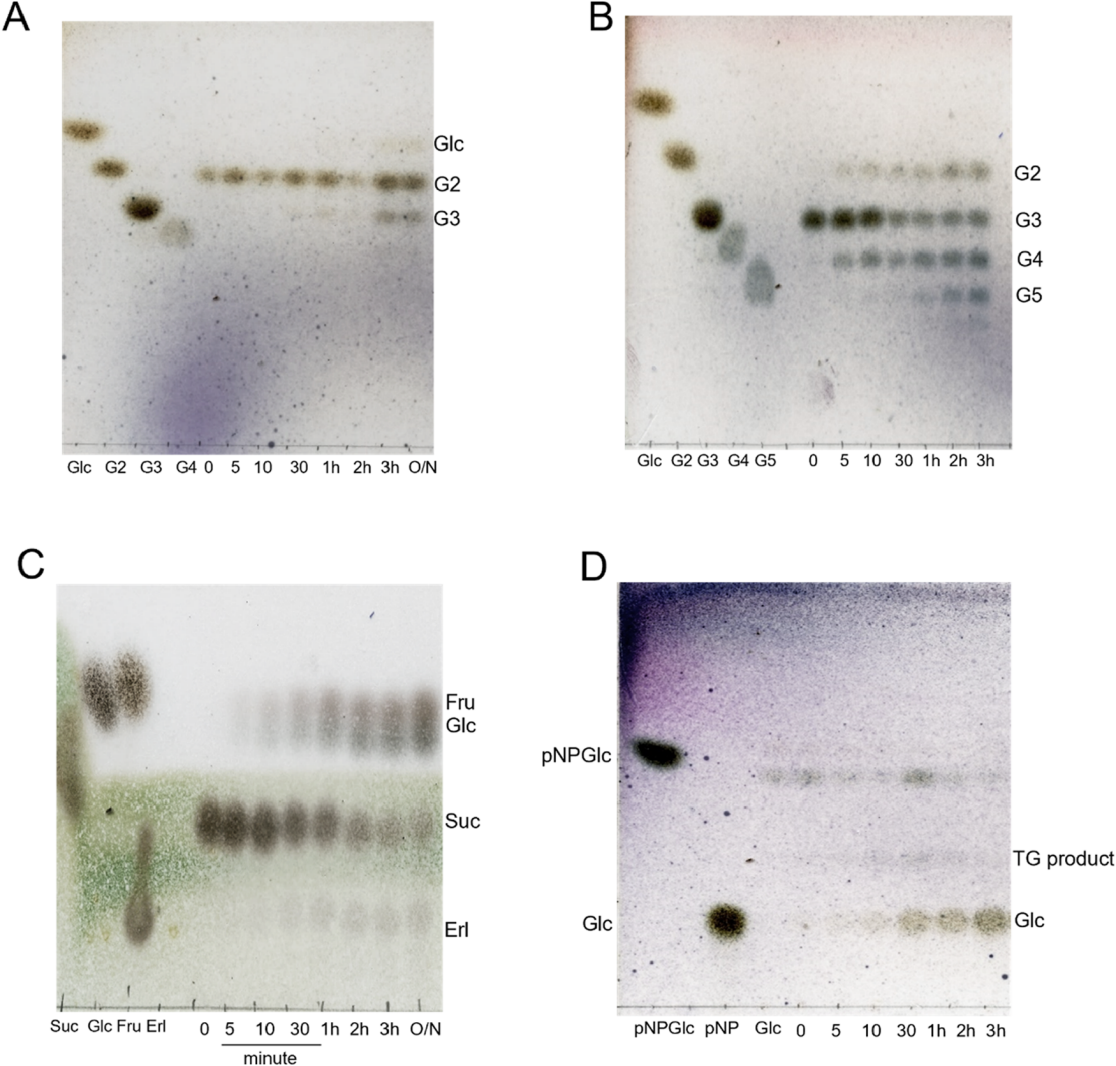
Time courses of reaction products from purified AaMalI analyzed by TLC. TLC analysis of reaction products generated by purified AaMalI (0.7 μm) was performed after incubation with (A) 0.5 M G2, (B) 0.5 M G3, (C) 0.5 M sucrose, and (D) 10 mM pNPGlc. Each reaction was incubated at 37 °C. Aliquots were taken at the indicated time points, and the reaction was stopped by heating. Five micrograms of total carbohydrate was loaded per lane. Erl, erlose; TG, putative transglucosylation product from pNPGlc.

### Characterization of AaMalI Pro222 and Tyr223 Mutants

Pro222 and Tyr223 of AaMalI, included in conserved region II, were substituted with Asn and His, respectively. These residues correspond to Pro226 and Tyr227 in HBG-III, which have been implicated in substrate preference differences among honeybee isozymes.^29^ Mutants (P222N and Y223H) and a double mutant (P222N and Y223H) were prepared as the wild type. From 250 mL of the culture supernatant, 0.38 mg of P222N (2.71 U/mg), 0.33 mg of Y223H (5.39 U/mg), and 0.35 mg of P222N/Y223H (3.20 U/mg) were obtained. These mutant enzymes presented a predominant band at 67 kDa after deglycosylation, similar to that of the wild type (Figure S1). In addition, several bands between 37 and 50 kDa were detected in the mutants, presumably due to partial deglycosylation or heterogeneous glycosylation at other sites.^36,37^ Based on the reaction rates toward various AGase, all mutants hydrolyzed sucrose and maltooligosaccharides more rapidly than did other natural substrates. Only in the double mutant P222N/Y223H, the reaction rates to isomaltose increased 68.3-fold compared to the wild type (Table S2). Reactions with predominant substrates, as analyzed on the predominant substrates in the wild-type enzyme, followed Michaelis–Menten kinetics, and the determined parameters are shown in Table 3 and Figure S2. The *k*_cat_/*K*_m_ values of both P222N and Y223H to sucrose were approximately 10% of that of the wild type, while those of G2 were 16.7% and 524%, respectively. Therefore, Y223H had an 8.3-fold higher preference (*k*_cat_/*K*_m_) for G2 over sucrose, whereas the wild type had a 5.4-fold higher preference for sucrose over G2, and P222N/Y223H had intermediate values between P222N and Y223H. In contrast to the wild type, which had a 112-fold higher *k*_cat_/*K*_m_ value for G3 than G2, the *k*_cat_/*K*_m_ values of P222N, Y223H, and P222N/Y223H for G3 were only 36.1-, 10.1-, and 35.0-fold, respectively. All of the mutant enzymes had *k*_cat_/*K*_m_ values to G4 that were approximately one-half of those to G3 as the wild type.

### Characterization of AaMalI A407E Mutant

Ala407 of AaMalI corresponds to Glu440 of *Bombyx mori* Sucrose hydrolase (BmSUH), a lepidopteran AGase involved in sucrose hydrolysis, in which this residue has been identified as a key determinant for sucrose specificity.^30^ A mutant enzyme, A407E (0.73 mg; 0.503 U/mg), was produced and purified as the wild type (Figure S1). The *k*_cat_/*K*_m_ values of A407E for sucrose and G2 were 36.5% and 65.2% of those of the wild type, respectively, indicating that this mutant enzyme maintained a preference for sucrose over G2. The *K*_m_ value for sucrose was approximately 2.12-fold lower than that of the wild type, whereas the relative *k*_cat_/*K*_m_ value decreased by about 2.74-fold. Among the maltooligosaccharides, A407E showed the highest *k*_cat_/*K*_m_ value for G3. However, the *k*_cat_/*K*_m_ value of A407E for G3 was only 47-fold higher than that for G2, whereas the *k*_cat_/*K*_m_ value of the wild type for G3 was 112-fold higher than that for G2.

### Structural Prediction of Wild-Type AaMalI and Its Mutants

The structural features of wild-type AaMalI and its mutants (P222N, Y223H, P222N/Y223H, and A407E) were analyzed using predicted models and reference ligand-bound structures of sucrose, G2, G3, and G4. AaMalI is predicted to be composed of four distinct domains, A, B, B’, and C (Figure S3). The overall fold of the mutant proteins was highly conserved compared to that of the wild-type enzyme. All four substrates were accommodated within the same binding pocket (Figure 3). The ligands were positioned in a conserved orientation with residues surrounding subsite −1 exhibiting a similar arrangement to those observed in other GH13 AGase. A salt bridge between Asp356 and Arg421 was observed, suggesting stabilization of the local conformation of the subsite −1 that accommodates the d-glucosyl residue. In addition, Asp76 formed a hydrogen bond with the 4-OH group of the glucosyl moiety, which may help stabilize its orientation and facilitate substrate recognition at subsite −1. Tyr79 exhibited stacking interactions with the glucosyl ring, which further contributed to substrate stabilization.

**Figure 3:**
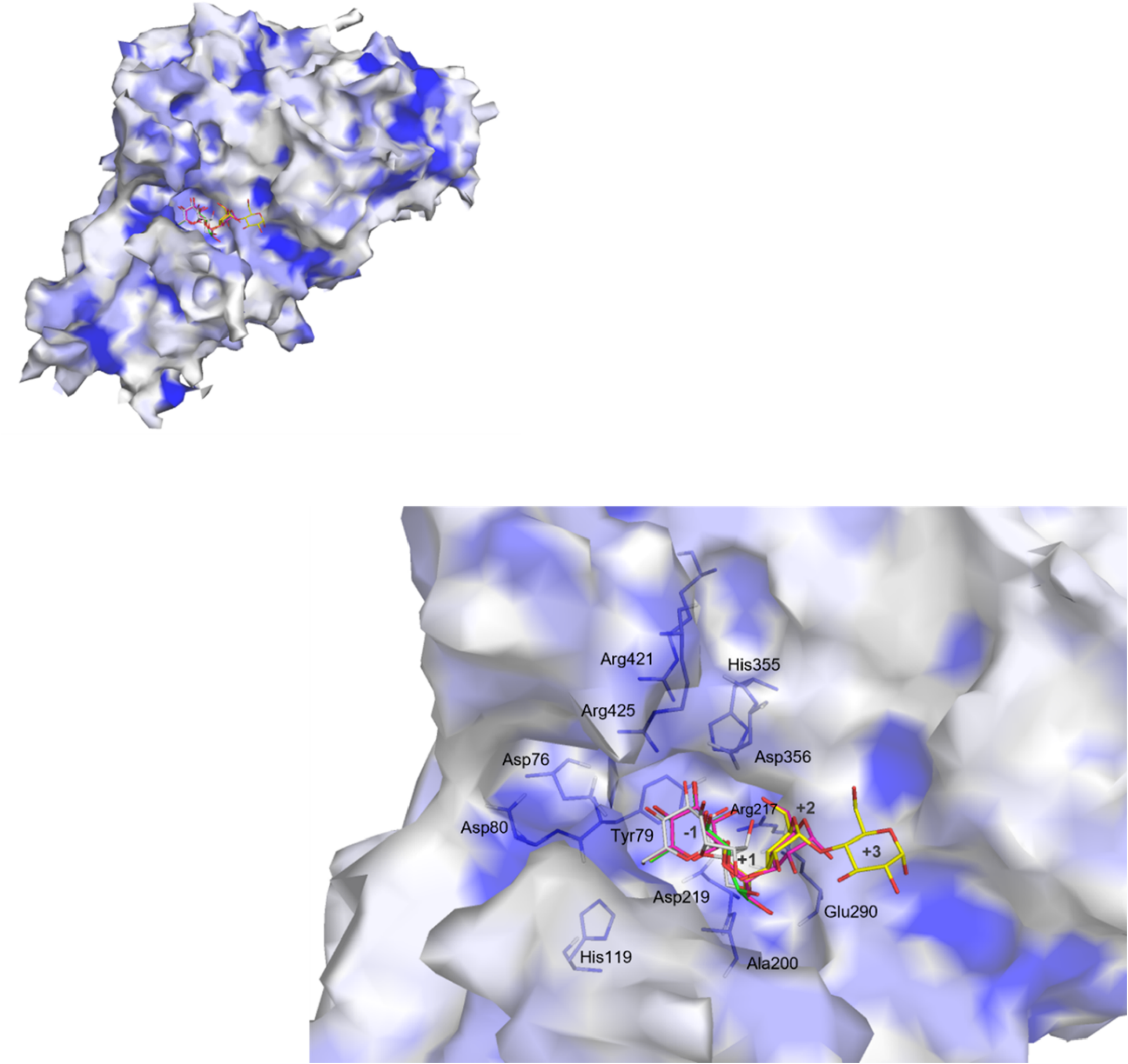
Surface representation and active site of AaMalI. The surface of AaMalI is colored based on hydrophobicity using the Kyte–Doolittle scale with a white-to-blue gradient. Blue indicates hydrophobic regions, while white/light-blue indicates hydrophilic regions. Superimposed ligand-bound structures with sucrose (PDB entry, 6LGG), G2 (PDB entry, 5ZCC), G3 (PDB entry, 5ZCD), and G4 (PDB entry, 5ZCE) are shown as sticks in white, green, magenta, and yellow, respectively. The conserved residues accommodated in the −1 subsite are represented as blue sticks. The subsites −1, +1, +2, and +3 are indicated. Key conserved residues surrounding the subsite −1 are labeled, highlighting their roles in substrate recognition and stabilization.

The sucrose binding structure of the wild type and mutants is described (Figure 4A–E). Tyr223 is positioned toward the fructosyl ring, exhibiting a CH–π interaction with 1-C of the fructosyl residue. Pro222, which is located adjacent to Tyr223, may contribute to shaping the local conformation of the binding pocket geometry by restricting backbone flexibility, thereby indirectly influencing substrate binding. Substitution of Tyr223 with His markedly reduced the specificity toward sucrose. Compared to Tyr, His223 possessed a smaller and more polar side chain and is not able to provide CH–π interaction with 1-C of fructosyl residue as Tyr does. Substitution of Pro222 with Asn altered the backbone rigidity adjacent to Tyr223 by increasing flexibility, resulting in the destabilization of the Michaelis complex (Figure 4B). In the A407E mutant, Glu407 was located outside the substrate-binding site and did not alter the positions of the substrate-binding residues, including Pro222 and Tyr223, consistent with retained sucrose preference. No direct contacts between Glu407 and sucrose were observed in the predicted structure (Figure 4E).

**Figure 4:**
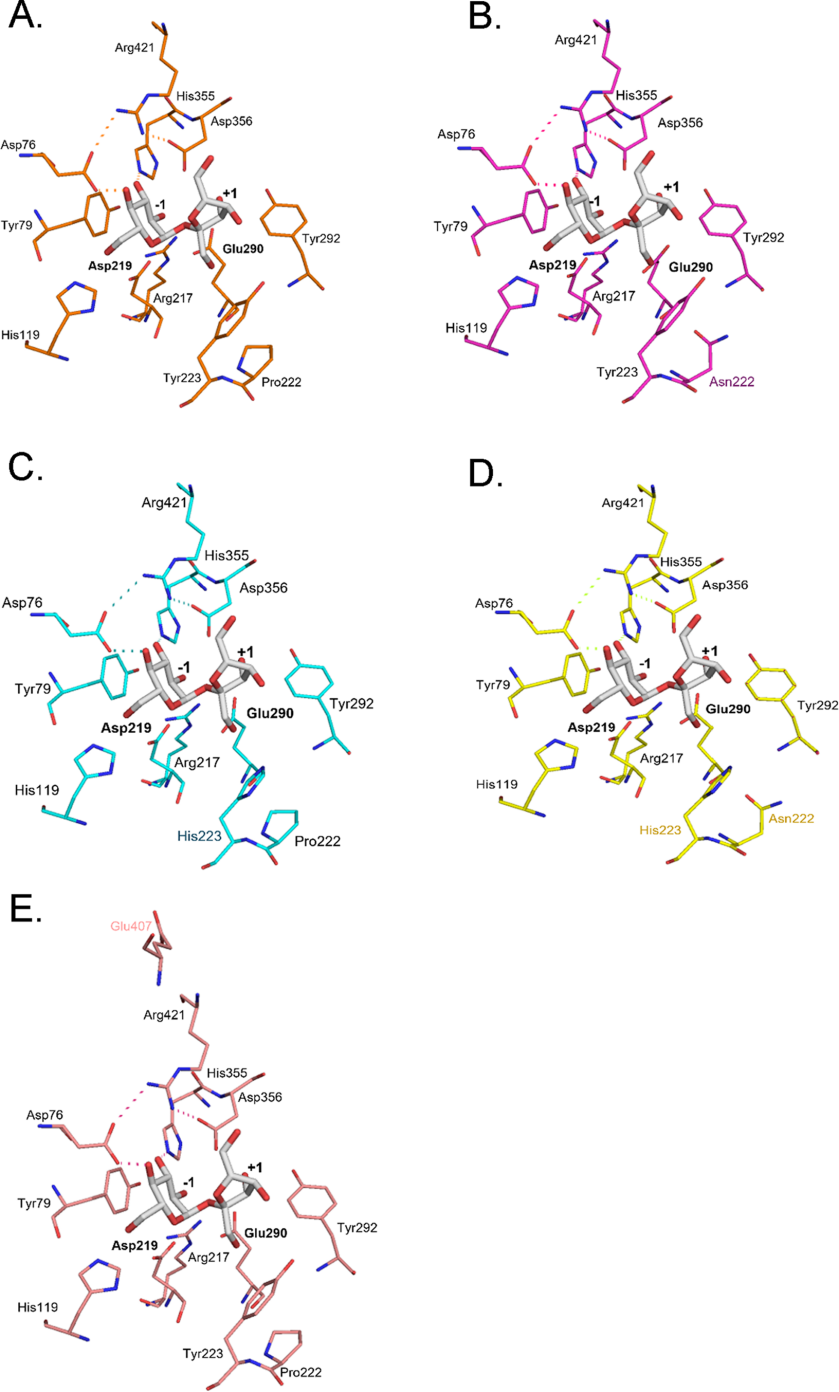
Structure comparison of the active site of wild-type AaMalI and its mutants in complex with sucrose. Close-up views of the substrate-binding pocket, highlighting key residues surrounding the subsites −1 and +1 are shown. Wild type (A), P222N (B), Y223H (C), P222N/Y223H (D), and A407E (E) are presented. Conserved residues and mutated residues are shown in stick representation. Asp219 and Glu290 act as nucleophile and catalytic acid/base, respectively. Hydrogen-bond interactions are indicated by dotted lines. Sucrose derived from PDB entry 6LGG is shown as gray sticks.

When the substrate was G2, the d-glucosyl residue at subsite +1 was positioned in proximity to Tyr292, which may contribute to orienting the glucosyl residue into an appropriate binding geometry (Figure 5A–E). Upon substitution of Tyr223 with His, the His residue at this position was oriented toward the d-glucosyl residue at the subsite +1 (Figure 5C,D), suggesting a potential role in substrate positioning rather than formation of a strong hydrogen bond. This structural feature was consistent with the enhanced catalytic efficiency toward G2 because the Y223H mutation significantly increased *k*_cat_/*K*_m_ for G2. His223 may also indirectly influence the local hydrogen bonding environment around the active site, including residues such as Tyr292. In addition, replacement of Tyr223 with His likely resulted in a slightly more open binding pocket due to the smaller side chain of the His residue, which may provide additional space for accommodation of the d-glucosyl moiety (Figure 6).

**Figure 5:**
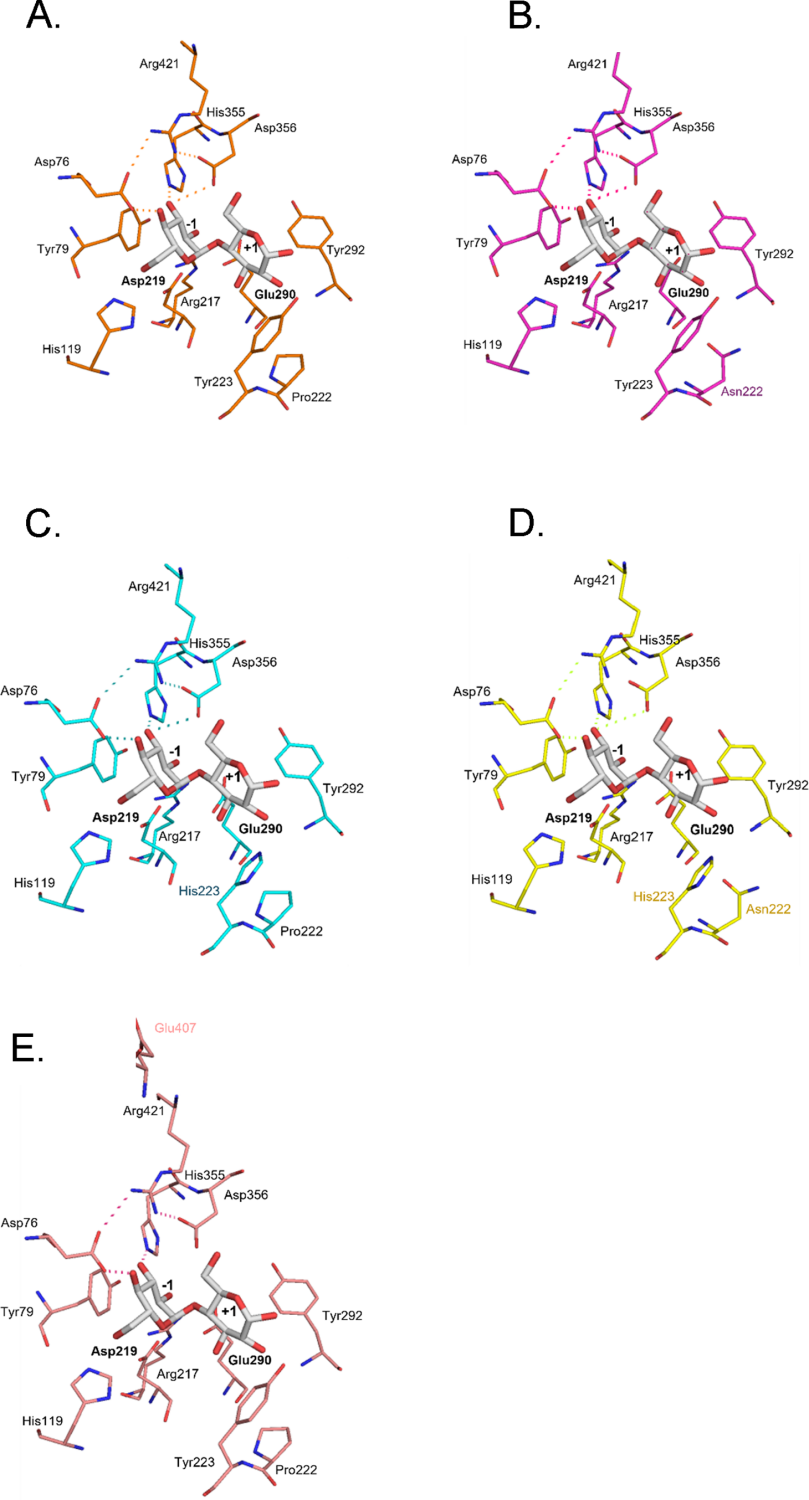
Structure comparison of the active site of wild-type AaMalI and its mutants in complex with G2. Close-up views of the substrate-binding pocket, highlighting key residues surrounding subsites −1 and +1 are shown. Wild type (A), P222N (B), Y223H (C), P222N/Y223H (D), and A407E (E) are presented. Conserved residues and mutated residues are shown in stick representation. Asp219 and Glu290 act as nucleophile and catalytic acid/base, respectively. Hydrogen-bond interactions are indicated by dotted lines. G2 derived from PDB entry 5ZCC is shown as gray sticks.

**Figure 6:**
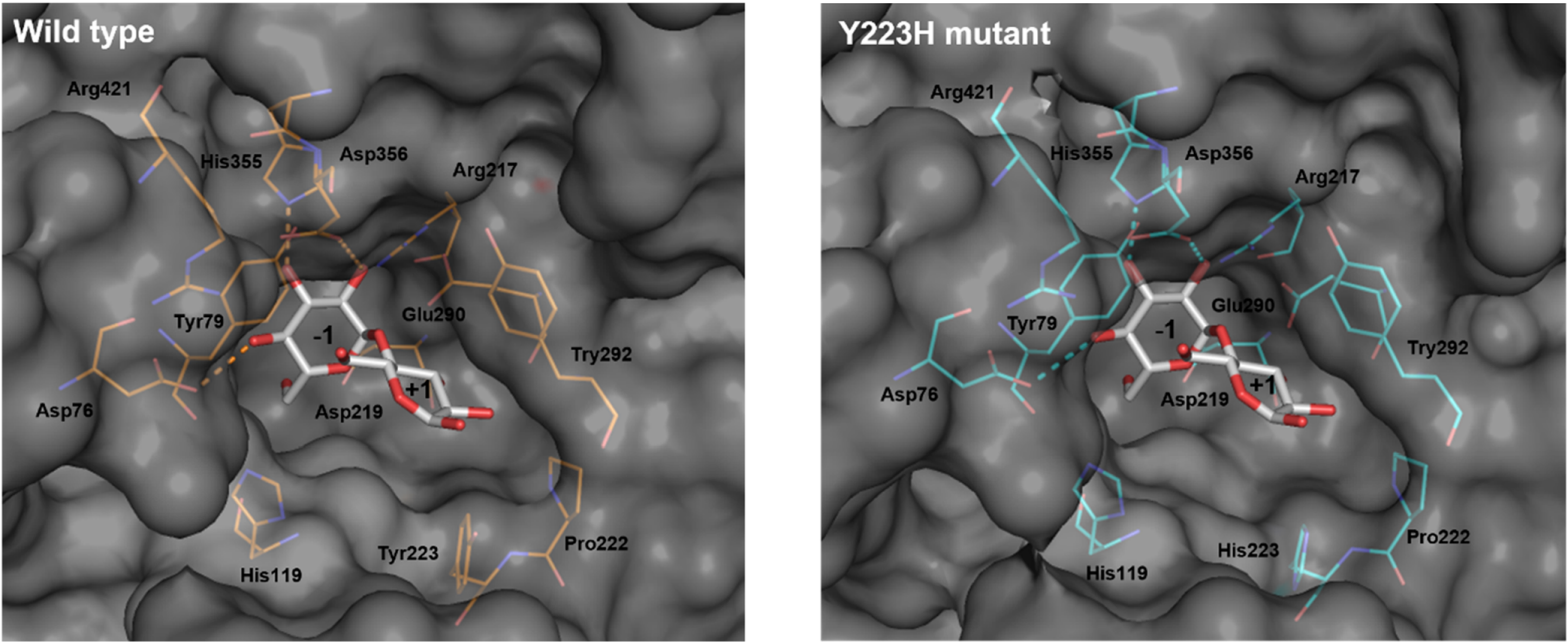
Comparison of the substrate-binding pockets of the wild-type and Y223H mutant in complex with G2. Close-up views of the substrate-binding pocket in the wild-type enzyme and the Y223H mutant bound to G2 are shown. Conserved residues in the subsite −1 and residue surrounding subsite +1 in stick representation. Dotted lines indicate hydrogen-bond interactions. G2 (PDB entry, 5ZCC) is shown as gray sticks.

In the G3 complex, Phe316 was positioned near subsite +2 and may contribute to stabilization of the glucosyl moiety through aromatic-sugar stacking interactions. The local conformation of this region appeared to be supported by a hydrogen bond involving the backbone of Ser320 (Figure S4). The binding mode of G4 was similar to that of G3, and no distinct additional interactions were observed beyond subsite +2 (Figure S5).

## Discussion

This study characterized salivary AGase from *Aedes aegypti* using the recombinant enzyme produced in the *K. phaffii* transformant. AaMalI was purified to homogeneity, as confirmed by SDS–PAGE, which showed a single band at 80 kDa. The AGase activity of AaMalI on pH and temperature using pNPGlc as substrate showed that the pH optimum of the enzyme was at pH 6.0, slightly lower than that reported for salivary AGase from other *Aedes* species.^17,18,38^ Generally, mosquito salivary AGases are stable and active in slightly acidic to neutral pH environments, facilitating effective sugar digestion during feeding.^18,26^ Such salivary activity likely contributes to rapid carbohydrate processing during nectar and blood feeding, consistent with the metabolic demands of the hematophagous insect. Both AGase from *C. quinquefasciatus* (CqMalI) and HBG-III retained more than 80% of AGase activity at moderate temperatures (35–40 °C).^19,27^ The optimal temperature in AaMalI was 40 °C, suggesting that it is physiologically relevant to blood feeding in humans. The partial multiple sequence alignment of AaMalI and other insects AGase showed that AaMalI is an orthologue of the AGase CqMalI and HBG-III, with high sequence identity (73% and 40%, respectively), as shown in Figure 7.

**Figure 7:**
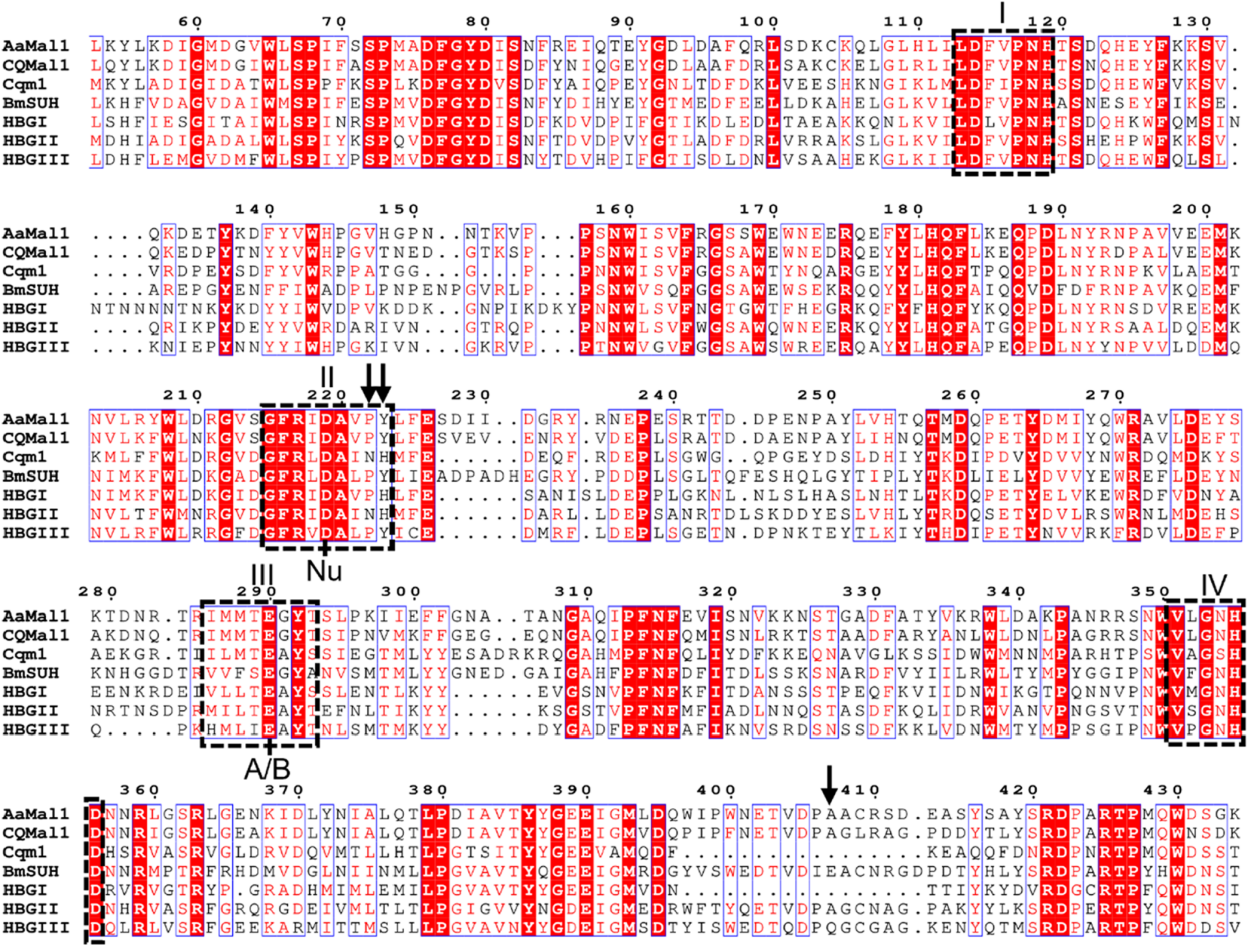
Partial amino-acid sequence alignment of GH13_17 AGase. Amino acid sequence alignment was constructed using MAFFTash. Sequences used were *Aa*MalI (Genbank AAA29352.1), *Culex quinoquefasciatus* maltase I (CqMalI, XP_001866573.2), binary toxin receptor protein from *Culex quinquefasciatus* (Cqm1, ASO96882.1), *Bombyx mori* Sucrose hydrolase (BmSUH, BAP18683.1), honeybee (*Apis mellifera*) AGase I (HBG-I, BAE86926.1), honeybee AGase II (HBG-II, BAE86927.1), and honeybee AGase III (HBG-III, BAA11466.1). Conserved regions are shown in boxes. Nu and A/B indicate the catalytic nucleophile and general acid/base catalysts, respectively. Arrows indicate mutation sites in the study.

The amino-acid residues that are key to substrate specificity in the conserved region II, as reported, are conserved.^29^ Insects’ AGases are mainly localized in the midgut and salivary gland,^15,39,40^ where carbohydrate digestion begins. Predominantly, AaMalI hydrolyzed sucrose with a high *k*_cat_ (193 s^–1^) and *K*_m_ (165 mM), similar to the high values also observed in HBG-III and CqMalI.^19,29^ The observed high *k*_cat_ and *K*_m_ values for sucrose hydrolysis may reflect the adaptation of insects’ AGase to efficiently process plant sap, in which sucrose concentrations typically range from several hundred millimolar to ∼1.4 M across herbaceous and tree species.^41^ This enzymatic adaptation is crucial for sustaining insect metabolism and survival, where the enzymes contribute to carbohydrate metabolism and energy balance.^42–44^ At higher substrate concentrations, AaMalI showed substrate inhibition and catalyzed transglucosylation reactions, as evidenced by the formation of erlose in the reaction with 0.5 M sucrose. Such transglucosylation activity is widely reported in microbial GH13 and GH31 AGase.^23,45–47^ In insects, this reaction participates in carbohydrate metabolism supporting insect survival and reproduction, particularly under starvation.^48^ Elevated substrate concentrations may induce osmotic stress, which, in turn, can negatively impact enzyme activity and reduce the overall reaction rate, as reported in the pea aphid (*Acrythosiphon pisum*).^49,50^ A putative transglucosylation product with pNPGlc, presumed to be pNP α-maltoside, was also detected by TLC at the later stage of incubation. However, confirmation of the specific linkages to these products would require further characterization, such as NMR and HPAEC-PAD, to confirm their identity. AaMalI appeared to catalyze α-(1→4)-glucosidic linkage formation through transglucosylation, in agreement with the activity reported for HBG-III. Consistent with this, α-(1→4)-linked products such as erlose and G3 were detected at 0.5 and 2 h from sucrose and G2, respectively. The high regioselectivity toward α-(1→4)-transglucosylation observed in AaMalI may be associated with the presence of a Pro residue at the position equivalent to Pro222, whereas the presence of Tyr (Tyr223 in AaMalI) is found mostly in GH13_18 enzymes acting on sucrose.^51,52^ AaMalI had the highest preference in G3, with little activity in the other glucobioses, except in maltooligosaccharides as commonly found in GH13 AGase.^2,53^

Although the presence of AGase has been reported in mosquitoes, no studies have confirmed the involvement of the region II residues in sucrose specificity for mosquito AGase. Mutational analysis revealed distinct effects on the catalytic efficiency and substrate preference among the AaMalI variants (P222N, Y223H, and P222N/Y223). All mutants had lower *k*_cat_/*K*_m_ values for sucrose than for G3 in the wild type. The single and double mutations in this region decreased the *k*_cat_ values for sucrose compared to those of the wild type. The introduction of His residue in two mutants (Y223H and P222/Y223H) greatly decreased *k*_cat_ for sucrose without any major change in *K*_m_ in AaMalI. This contrasts with HBG-III mutants, where both *K*_m_ and *k*_cat_ were reported to be lower.^29^ In the single Y223H mutation, the *k*_cat_/*K*_m_ value for G2 was 5.23-fold higher than that of the wild type due to an increased *k*_cat_ value. A slight but not significant increase in the *K*_m_ values for maltoglycosaccharides was observed in this mutant. Nevertheless, the presence of the His residue appears to confer specificity for α-(1→4)-glucosidic linkage, as commonly observed in many GH13 α-(1→4)-specific enzymes, such as α-amylase, cyclodextrin glucanotransferases, neopullulanasess, and AGases.^12,13,54^ In contrast, the mutation at Pro222 in AaMalI resulted in a lower substrate binding affinity for maltooligosaccharides, as demonstrated by reduced *k*_cat_ and increased *K*_m_ values. The presence of Asn and His in HBG-II at the equivalent position in AaMalI enables the hydrolysis of broad glucobioses with α-1,2, α-1,3, and α-1,6-glucosidic linkage and sucrose besides maltooligosaccharides.^55^ Aside from hydrolytic activity, HBG-II also exhibits transglucosylation, generating panose (α-d-Glc*p*-(1→6)-α-d-Glc*p*-(1→4)-α-d-Glc*p*) and theanderose (α-d-Glc*p*-(1→6)-α-d-Glc*p*-(1↔2)-β-d-Fruc*f*) from maltose and sucrose as substrates.^29^ An increase in isomaltase activity was observed in the P222N/Y223H mutant. However, further studies are necessary on hydrolysis and transglucosylation in the P222N/Y223H mutant.

Structural prediction of AaMalI provided insights into the observed kinetic behavior. Overall structural modeling indicated that the overall fold of the AaMalI mutants was conserved. However, minor conformational differences relative to the wild-type enzyme may influence the substrate preference. Both AaMalI and its mutants showed a preference for maltooligosaccharides such as G3 and G4, which were longer than G2. This preference may be associated with Tyr292, which is located near the subsite +2 and may help to stabilize the d-glucosyl unit. Aromatic stacking interactions between Tyr292 and Phe316 may help to precisely position the spatial position of Tyr292 in the binding affinity toward trisaccharides in AaMalI. A similar mechanism has been reported for trisaccharide specificity in *Streptococcus mutans* dextran glucosidase (SmDG), in which aromatic residues of Trp238 and Tyr262 contribute to substrate recognition.^56,57^

In the G2-bound complex, substitution of Tyr223 with His might result in more favorable interactions with the glucosyl residue of maltooligosaccharides in subsite +1.

Notably, many GH13 with specificity toward α-(1→4)-linked substrates possess a histidine residue at the corresponding position. This histidine has been reported to form a hydrogen bond with the d-glucosyl residue in subsite +1, thereby enhancing G2 specificity.^12,53^ This interpretation is consistent with the observations for the Y223H and P222N/Y223H mutants. This substitution may also reorganize the local interaction network and indirectly influence Tyr292 through water-mediated contacts. Given the proton-transfer capability of histidine, this substitution may modulate the local electrostatic environment. Furthermore, our experimental observations are consistent with molecular dynamics simulations reported for HBG-III,^58^ in which van der Waals contacts involving Tyr227 (corresponding to Tyr223 in AaMalI) were suggested to contribute substantially to substrate preference. Replacement of Tyr with a more polar His reduced van der Waals contributions and introduced weak polar and electrostatic interactions, thereby favoring G2 binding. Overall, substitution of a bulky aromatic residue with histidine is likely to alter the active-site geometry and modulate the substrate preference.

In homologous GH13_17 AGase in Lepidoterans with high sucrose specificity, such as BmSUH, residues Gln191, Tyr251, and Glu440 (corresponding to Val163, Tyr223, and Ala407 in AaMalI) have been reported to be involved in substrate recognition.^30^ Glu440 in BmSUH directly interacts with the fructosyl residue at the +1 subsite. However, replacing Ala407 with Glu in AaMalI is unlikely to play a major role in sucrose binding, as no additional interactions with the sucrose ligand were observed. This difference may reflect the unique catalytic characteristics of sucrose hydrolase.

In recent years, increasing attention has been given to mosquito salivary proteins as potential targets for interrupting parasite transmission.^59,60^ Among them, apyrase is one of the most studied enzymes, abundantly secreted in mosquito saliva that facilitates blood feeding by inhibiting platelet aggregation and coagulation,^61,62^ and also contributes to *Plasmodium* development by modulating hemostasis and immune responses within the blood bolus.^63^ Given these biological roles, RNA interference (RNAi) has emerged as an effective molecular strategy for mosquito control. Several studies have utilized RNAi-mediated to silence mosquito genes at developmental stages.^64^ RNAi-mediated silencing of insecticide-resistance genes, including *Abcg*4 transporter and voltage-gated sodium channels, has been shown to increase the susceptibility of mosquitoes to pyrethroid insecticides.^65,66^ Beyond RNAi-based strategies, gene-editing approaches have also been applied to control arbovirus transmission. *Maltase 1 (MAL1)*, targeted by CRISPR/Cas9 knockout, significantly reduced the level of DENV2 replication in the *Aedes aegypti* midgut. *MAL1* deficiency decreased hatching and pupation rates and shortened female lifespan although mosquitoes remained viable and capable of reproduction.^67^ These findings identify *MAL1* as a promising genetic target for disrupting virus transmission and mosquito development.

Upon these findings, AGase represents an interesting enzyme for further exploration in mosquito metabolism and pathogen interaction. Although this study primarily focused on characterizing substrate specificity, the observed alteration in enzymatic preference from sucrose to G2 may provide preliminary insight into carbohydrate utilization during mosquito feeding.

## Conclusions

Sucrose is a major energy source for insects, including *Aedes aegypti*. Here, we identified residues involved in sucrose metabolism in the salivary AGase (AaMalI) of *Ae. aegypti*. Salivary AaMalI catalyzed the hydrolysis of sucrose and G2 and exhibited substrate preferences similar to those of insect GH13_17 AGase, such as CqMalI and HBG-III. A mutation in the conserved region II motif (DAxPY) altered substrate specificity, where substitution with His increased the affinity toward G2. By linking structure changes to substrate adaptation, this study provides mechanistic insight into the carbohydrate metabolism in mosquito saliva. The results highlight AGase as a potential metabolic target for future enzyme inhibition and knockdown studies to assess its role and that of its orthologs.

## Supplementary Material



## Data Availability

All data supporting the findings of this study are included within the manuscript and its Supporting Information files.

## Notes

Tables S1 and S2, Figures S1–S5, and additional experimental details are provided. (PDF)

## References

[1] Kimura A. (2000). Molecular
anatomy of α-glucosidase. Trends Glycosci.
Glycotechnol..

[2] Okuyama M., Saburi W., Mori H., Kimura A. (2016). α-Glucosidases
and α-1,4-glucan lyases: structures, functions, and physiological
actions. Cell. Mol. Life Sci..

[3] Stanley D., Rejzek M., Naested H., Smedley M., Otero S., Fahy B., Thorpe F., Nash R. J., Harwood W., Svensson B., Denyer K., Field R. A., Smith A. M. (2011). The role
of alpha-glucosidase in germinating barley grains. Plant Physiol..

[4] Zhai X., Wu K., Ji R., Zhao Y., Lu J., Yu Z., Xu X., Hang J. (2022). Structure and function insight of the α-glucosidase
QsGH13 from *Qipengyuania seohaensis* sp. SW-135. Front. Microbiol..

[5] Rengarajan S., Palanivel R. (2024). Purification,
characterization of novel α-glucosidase
from Debaryomyces hansenii strain MCC 0202 and chromatographic separation
for high purity isomaltoligosaccharides production. Process Biochem..

[6] Cuebas-Irizarry M., Irrizarry-Caro R. A., López-Morales C., Badillo-Rivera K. M., Rodríguez-Minguela C. M., Montalvo-Rodríguez R. (2017). Cloning and
molecular characterization of an alpha-glucosidase (MalH) from the
halophilic archaeon *Haloquadratum walsbyi*. Life.

[7] da
Silva T. M., Michelin M., de Lima Damásio A. R., Maller A., Almeida F. B. D. R., Ruller R., Ward R. J., Rosa J. C., Jorge J. A., Terenzi H. F., de Moraes
Polizali M. (2009). Purification and biochemical characterization of a
novel α-glucosidase from *Aspergillus niveus*. Antonie van Leeuwenhoek.

[8] Janeček Š., Svensson B. (2022). How many α-amylase
GH families are there in the
CAZy database?. Amylase.

[9] Stam M. R., Danchin E. G., Rancurel C., Coutinho P. M., Henrissat B. (2006). Dividing the
large glycoside hydrolase family 13 into subfamilies: Towards improved
functional annotations of α-amylase-related proteins. Protein Eng. Des. Sel..

[10] Janeček Š. (2023). Advances
in Amylases—What’s Going on?. Molecules.

[11] Møller M. S., Fredslund F., Majumder A., Nakai H., Poulsen J. C. N., Leggio L.Lo., Svensson B., Abou Hachem M. (2012). Enzymology
and structure of the GH13_31 glucan 1,7-α-glucosidae that confers
isomaltooligosaccharide utilization in the probiotic *Lactobacillus
acidophilus* NCFM. J. Bacteriol..

[12] Auiewiriyanukul W., Saburi W., Kato K., Yao M., Mori H. (2018). Function and
structure of GH13_31 α-glucosidase with high α-(1→4)-glucosidic
linkage specificity and transglucosylation activity. FEBS Lett..

[13] MacGregor E. A., Janeček Š., Svensson B. (2001). Relationship of sequence
and structure to specificity in the α-amylase family of enzymes. Biochim. Biophys. Acta.

[14] Chiba, S. ; Minamiura, N. α-Glucosidases. In Handbook of Amylases and Related Enzymes: Their Sources, Isolation Methods, Properties and Applications; Pergamon: Oxford, U.K., 1988; pp 104–116.

[15] da
Costa S. G., Bates P., Dillon R., Genta F. A. (2019). Characterization
of α-glucosidase from *Lutzomyia longipalpis* reveals independent hydrolysis systems for plant blood sugars. Front. Physiol..

[16] Gary
Jr R. E., Foster W. A. (2001). Effects of available sugar on the
reproductive and vectorial capacity of the malaria vector *Anopheles gambiae* (Diptera: Culicidae). J. Med. Entomol..

[17] James A. A., Blackmer K., Racioppi J. V. (1989). A salivary gland-specific,
maltase-like
gene of the vector mosquito, *Aedes aegypti*. Gene.

[18] Marinotti O., de Brito M., Moreira C. K. (1996). Apyrase and α-glucosidase in
the salivary glands of *Aedes albopictus*. Comp. Biochem. Physiol. B: Biochem. Mol. Biol..

[19] Suthangkornkul R., Sirichaiyakul P., Sungvornyothin S., Thepouyporn A., Svasti J., Arthan D. (2015). Functional
expression and molecular
characterization of *Culex quinquefasciatus* salivary
α-glucosidase (MalI). Protein Expr. Purif..

[20] Jariyapan N., Choochote W., Jitpakdi A., Harnnoi T., Siriyasatein P., Wilkinson M. C., Junkum A., Bates P. A. (2007). Salivary gland proteins
of the human malaria vector, *Anopheles dirus* B (Diptera:
Culicidae). Rev. Inst. Med. Trop. Sao Paulo.

[21] Schmitt A. (2014). The invisible
communities of nectar: How yeast and bacteria alter nectar characteristics. Duluth J. Undergrad. Biol..

[22] Fenner E. D., Scapini T., da Costa Diniz M., Giehl A., Treichel H., Álvarez-Pérez S., Alves S. L. (2022). Nature’s Most Fruitful Threesome: The Relationship between
Yeasts, Insects, and Angiosperms. J. Fungi.

[23] Ernits K., Kjeldsen C., Persson K., Grigor E., Alamäe T., Visnapuu T. (2021). Structural Insight
into a Yeast Maltase—The *Ba*AG2 from *Blastobotrys adeninivorans* with
Transglycosylating Activity. J. Fungi.

[24] Bissaro B., Monsan P., Fauré R., O’Donohue M. J. (2015). Glycosynthesis
in a waterworld: new insight into the molecular basis of transglycosylation
in retaining glycoside hydrolases. Biochem.
J..

[25] Merdzo Z., Narmontaite E., Gonzalez-Alfonso J. L., Poveda A., Jimenez-Barbero J., Plou F. J., Fernández-Lobato M. (2024). Insights into the transglucosylation
activity of α-glucosidase from *Schwanniomyces occidentalis*. Appl. Microbiol. Biotechnol..

[26] Williams A. E., Gittis A. G., Botello K., Cruz P., Martin-Martin I., Valenzuela Leon P. C., Leon P. C. V., Sumner B., Summer B., Bonilla B. (2024). Structural
and functional comparisons of salivary α-glucosidases
from the mosquito vectors *Aedes aegypti*, *Anopheles gambiae*, and *Culex quinquefasciatus*. Insect Biochem. Mol. Biol..

[27] Nishimoto M., Kubota M., Tsuji M., Mori H., Kimura A., Matsui H., Chiba S. (2001). Purification and Substrate
Specificity
of Honeybee, *Apis mellifera* L., α-Glucosidase
III. Biosci. Biotechnol. Biochem..

[28] Kubota M., Tsuji M., Nishimoto M., Wongchawalit J., Okuyama M., Mori H., Matsui H., Surarit R., Svasti J., Kimura A., Chiba S. (2004). Localization
of α-glucosidases
I, II, III in organs of European honeybees, *Apis mellifera* L., and the origin of α-glucosidase in honey. Biosci. Biotechnol. Biochem..

[29] Ngiwsara L., Iwai G., Tagami T., Sato N., Nakai H., Okuyama M., Mori H., Kimura A. (2012). Amino acids in conserved
region II are crucial to substrate specificity, reaction velocity,
and regioselectivity in the transglycosylation of honeybee GH-13 α-glucosidases. Biosci. Biotechnol. Biochem..

[30] Miyazaki T., Park E. Y. (2020). Structure–function
analysis of silkworm sucrose
hydrolase uncovers the mechanism of substrate specificity in GH13
subfamily 17 exo-α-glucosidases. J. Biol.
Chem..

[31] Gupta R., Brunak S. (2002). Prediction of glycosylation across the human proteome
and the correlation to protein function. Pac.
Symp. Biocomput..

[32] Laemmli U. K. (1970). Cleavage
of structural proteins during the assembly of the head of bacteriophage
T4. Nature.

[33] Bradford M. M. (1976). A rapid
and sensitive method for the quantitation of microorganism quantities
of protein utilizing the principle of protein-dye binding. Anal. Biochem..

[34] Abramson J., Adler J., Dunger J., Richard E., Green T., Pritzel A., Ronneberger O., Willmore L., Ballard A. J., Bambrick J., Bodensteein S. W., Evans D. A., Hung C.-C., O’Neil M., Reiman D., Tunyasuvunakool K., Wu Z., Žemgulyte A., Arvaniti E., Beattie C. (2024). Accurate structure prediction
of biomolecular interactions with AlphaFold
3. Nature.

[35] Schödinger, LCCThe Pymol Molecular Graphics System, Version 2.5; Schrödinger, LLC: New York, 2021.

[36] Daly R., Hearn M. T. W. (2005). Expression of
heterologous proteins in *Pichia
pastoris*: a useful experimental tool in protein engineering
and production. J. Mol. Recognit..

[37] Yamaguchi S., Sunagawa N., Tachioka M., Igarashi K., Samejima M. (2020). Thermostable
mutants of glycoside hydrolase family 6 cellobiohydrolase from the
basidiomycete *Phanerochaete chrysosporium*. J. Appl. Glycosci..

[38] Marinotti O., James A. (1990). An α-glucosidase in the salivary
glands of the vector mosquito, *Aedes aegypti*. Insect Biochem..

[39] Asadi A., Ghadanyari M., Sajedi R. H., Sendi J. J., Tabari M. (2012). Biochemical
characterization of α-and β-glucosidases in alimentary
canal, salivary glands and haemolymph of the rice green caterpillar, *Naranga aenescens* M. (Lepidoptera: Noctuidae). Biologia.

[40] Wada-Katsumata A., Schal C. (2021). Salivary digestion
extends the range of sugar-aversions in the German
cockroach. Insects.

[41] Broussard L., Abadie C., Lalande J., Limami A. M., Lothier J., Tcherkez G. (2023). Pholem sap composition: what have we learnt from metabolomics?. Int. J. Mol. Sci..

[42] Nayar J. K., Sauerman D. M. (1971). The effect of diet on life-span, fecundity and flight
potential of *Aedes teaniorhynchus* adults. J. Med. Entomol..

[43] Zhou G., Pennington J., Wells M. A. (2004). Utilization of pre-existing energy
stores of female *Aedes aegypti* mosquitoes during
the first gonotropic cycle. Insect Biochem.
Mol. Biol..

[44] Kishimoto H., Adachi I. (2010). Effects of sucrose on survival and ovisposition of
the predacious insects *Stethorus japonicus* (Coleoptera:
Coccinellidae), *Oligota kashmirica benefica* (Coleoptera:
Staphylinidae), and *Scolothrips takahashii* (Thysanoptera:
Thripidae). Appl. Entomol..

[45] Casa-Villegas M., Marín-Navarro J., Polaina J. (2017). Synthesis of isomaltooligosaccharides
by *Saccharomyces cerevisiae* cells expressing *Asperigillus niger* α-glucosidase. ACS Omega.

[46] Chen H., Yang S., Xu A., Jiang R., Tang Z., Wu J., Zhu L., Liu S. (2019). Insight into the glycosylation
and hydrolysis kinetics of alpha-glucosidase in the synthesis of glycosides. Appl. Microbiol. Biotechnol..

[47] Merdzo Z., Narmontaite E., Gonzalez-Alfonso J., Poveda A., Jimenez-Barbero J., Plou F. J., Fernández-Lobato M. (2024). Insight into
transglucosylation
activity of α-glucosidase from *Schwaniomyces occidentalis*. Appl. Microbiol. Biotechnol..

[48] Arrese E. L., Soulages J. L. (2010). Insect fat body:
energy, metabolism, and regulation. Annu. Rev.
Entomol..

[49] Ashford D. A., Smith W. A., Douglas A. E. (2000). Living on a high sugar diet: the
fate of sucrose ingested by a phloem-feeding insect, the pea aphid *Acrythosiphon pisum*. J. Insect Physiol..

[50] Karley A. J., Ashford D. A., Minto L. M., Pritchard J., Douglas A. E. (2005). The significance of gut sucrase activity
for osmoregulation
in the pea aphid, *Acrythosiphon pisum*. J. Insect Physiol..

[51] Franceus J., Capra N., Desmet T., Thunnissen A- M. W. H. (2019). Structural
comparison of a promiscuous and highly specific sucrose 6F-phospahte
phosphorylase. Int. J. Mol. Sci..

[52] Franceus J., Desmet T. (2020). Sucrose phosphorylase
and related enzymes in glycoside
hydrolase family 13: discovery, application and engineering. Int. J. Mol. Sci..

[53] Wangpaiboon K., Laohawuttichai P., Kim S. Y., Mori T., Nakapong S., Pichyangkura R., Pongsawasdi P., Hakoshima T., Krusong K. (2021). A GH13 α-glucosidase
from *Weissella cibaria* uncommonly acts on short-chain
maltooligosaccharides. Acta. Crystallogr., Sect.
D: Struct. Biol..

[54] Hung V. S., Hatada Y., Goda S., Lu J., Hidaka Y., Li Z., Akita M., Ohta Y., Watanabe K., Matsui H. (2005). α-Glucosidase
from a strain of deep-sea Geobacillus: a potential
enzyme for the biosynthesis of complex carbohydrates. Appl. Microbiol. Biotechnol..

[55] Takewaki S.-i., Kimura A., Kubota M., Chiba S. (1993). Substrate specificity
and subsite affinities of honeybee α-glucosidase II. Biosci. Biotechnol. Biochem..

[56] Saburi W., Mori H., Saito S., Okuyama M., Kimura A. (2006). Structural
elements in dextran glucosidase responsible for high specificity to
long chain substrate. Biochem. Biophys. Acta.

[57] Kobayashi M., Saburi M., Nakatsuka D., Hondoh H., Kato K., Okuyama O., Mori H., Kimura A., Yao M. (2015). Structural
insisght into the catalytic reaction that is involved in the reorientation
of Trp238 at the substrate-binding site in GH13 dextran glucosidase. FEBS Lett..

[58] Na
Ayutthaya P. P., Chanchao C., Chunsrivirot S. (2018). Insight into
the substrate specificity change caused by the Y227H mutation of α-glucosidase
III from the European honeybee (*Apis mellifera*) through
molecular dynamics simulations. PLoS One.

[59] Klug D., Gautier A., Calvo E., Marois E., Blandin S. A. (2023). The salivary
protein Saglin facilitates efficient midgut colonization of *Anopheles* mosquitoes by malaria parasites. PLoS Pathog..

[60] Martin-Martin I., Smith L. B., Chagas A. C., Sá-Nunes A., Shrivastava G., Valenzuela-Leon P. C., Calvo E. (2020). *Aedes albopictus* D7 Salivary Protein Prevents Host
Hemostasis and Inflammation. Biomolecules.

[61] Champagne D. E., Smartt C. T., Riberio J. M., James A. A. (1995). The salivary gland-specific
apyrase of the mosquito *Aedes aegypti* is a member
of the 5′-nucleotidase family. Proc.
Natl. Acad. Sci. U.S.A..

[62] Sun D., McNicol A’., James A. A., peng Z. (2006). Expression of functional
recombinant mosquito salivary apyrase: a potential therapeutic platelet
aggregation inhibitor. Platelets.

[63] Pala Z. R., Alves E. S. T. L., Minai M., Crews B., Patino-Martinez E., Carmona-Rivera C., Valenzuela Leon P. C., Martin-Martin I., Flores-Garcia Y., Cachau R. E., Muslinkina L., Gittis A. G., Srivastava N., Garboczi D. N., Alves D. A., Kaplan M. J., Fischer E., Calvo E., Vega-Rodriguez J. (2024). Mosquito salivary
apyrase regulates blood meal hemostasis and facilitates malaria parasite
transmission. Nat. Commun..

[64] Yadav M., Dahiya N., Sehrawat N. (2023). Mosquito gene targeted RNAi studies
for vector control. Funct. Integr. Genomics.

[65] Negri A., Ferrari M., Nodari R., Coppa E., Mastrantonio V., Zanzani S., Porretta D., Bandi C., Urbanelli S., Epis S. (2019). Gene silencing through
RNAi and antisense Vivo-Morpholino increases
the efficacy of pyrethroids on larvae of *Anopheles stephensi*. Malar. J..

[66] Bona A. C. D., Chitolina R. F., Fermino M. L., de Castro
Poncio L., Weiss A., Lima J. B. P., Paldi N., Bernaardes E. S., Henen J., Maori E. (2016). Larval application
of sodium channel
homologous dsRNA restores pyrethroid insecticide susceptibility in
a resistant adult mosquito population. Parasites
Vectors.

[67] Li M. J., Cheng J., Zou Y. M., Liu Q. C., Zhu D., Lan C. J. (2025). Maltase 1 regulates
DENV2 infection and life history
in *Aedes aegypti*. Insect Sci..

