# Supplementary material for: Functional Analysis of Amino Acid Residues Responsible for Substrate Specificity of GH13_17 α‑Glucosidase from Aedes aegypti Saliva (AaMalI)
Source: ACS Omega. 2026 Mar 2;11(10):15795–808. doi: 10.1021/acsomega.5c08180 (PMC13000629; doi:10.1021/acsomega.5c08180)
Supplement: Supplementary file 1 [file ao5c08180_si_001.pdf]

Supporting Information

Functional analysis of amino acid residues responsible for substrate specificity of GH13_17 α-glucosidase from *Aedes aegypti* saliva (AaMalI)

Waraporn Auiewiriyanukul[1,*], *Wataru Saburi*[2], *Haruhide Mori*[2], *Dumrongkiet Arthan*[3] *Sorachat Tharamak*[4]

[1] Department of Biochemistry, Faculty of Science, Kasetsart University, 50 Ngamwongwan, Chatuchak, Bangkok, Thailand

[2] Research Faculty of Agriculture, Hokkaido University, Kita 9 Nishi 9, Sapporo, Japan

[3] Department of Tropical Nutrition and Food Science, Faculty of Tropical Medicine, Mahidol University, Bangkok, Thailand

[4] Department of Chemistry and Center of Excellence for Innovation in Chemistry, Special Research Unit for Advanced Magnetic Resonance, Faculty of Science, Kasetsart University, 50 Ngamwongwan, Chatuchak, Bangkok, Thailand

[*] Corresponding author E-mail: waraporn.au@ku.th

Contents

Table S1. Primer sequences used for construction AaMalI expression plasmid and mutants

Table S2. Reaction rate of AaMalI mutants with various substrates

Figure S1. SDS-PAGE analysis of purified AaMalI and its mutants

Figure S2. Kinetic analysis of reaction of AaMalI wild-type (WT) and mutant enzymes

Figure S3. The overall structure of AaMalI

Figure S4. Structure comparison of the active site of wild-type AaMalI and its mutants in complex with G3

Figure S5. Structure comparison of the active site of wild-type AaMalI and its mutants in complex with G4

Table S1. Primer sequences used for construction AaMalI expression plasmid and mutants.

**Primer names**	**Sequences (5'→ 3')**	**Mutation site**	**Note**
AaMalI_F	ATGAAGATCTTTGTTCCACTTCTAAGC	-	For wild-type construct
AaMalI_R	TTAAGA AACAATCGGGTTCTT TCCCC	-	For wild-type construct
AaEcoRI-F	GGGGGG*GAATTC*TTGGACTGGTGGGAACAT GGAAAC	-	For wild-type construct with EcoRI site
AaHisNotI-R	GGGGGG*GCGGCCGC*TAATGATGATGATGAT GATGAGAAACAATCGGGTTCTTTC	-	For wild-type construct with NotI site
P222N_F	GCAGTA*AAC*TATCTTTTCGAGAGCGAC	Pro222 → Asn	For P222N mutant
P222N_R	CTCGAAAAGATA*GTT*TACTGCATCGAT	Pro222 → Asn	For P222N mutant
Y223H_F	GTACCG*CAT*CTTTTCGAGAGCGACATT	Tyr223 → His	For Y223H mutant
Y223H_R	AATGTCGCTCTCGAAAAG*ATG*CGGTAC	Tyr223 → His	For Y223H mutant
P222N/Y223H_F	GTA*AACCAT*CTTTTCGAGAGCGACATT	Pro222/Tyr223→Asn222/His223	For P222N/Y223H mutant
P222N/Y223H_R	GAAAAG*ATGGTT*TACTGCATCGATTCT	Pro222/Tyr223 → Asn222/His223	For P222N/Y223H mutant
A407E_F	GATCCG*GAA*GCTTGCAGATCAGATGAG	Ala407 → Glu	For A407E mutant
A407E_R	GCAAGC*TTC*CGGATCAACAGTTTCGTT	Ala407 → Glu	For A407E mutant

The restriction site and mutation site are shown in italics.

Table S2. Reaction rate of AaMalI mutants with various substrates.

Substrate	Rate (s^{-1})			
	**P222N**	**Y223H**	**P222N/Y223H**	**A407E**
Sucrose	1.01 ± 0.05	0.82 ± 0.03	0.11 ± 0.004	0.87 ± 0.05
G2	0.07 ± 0.006	1.62 ± 0.21	0.392 ± 0.01	0.055 ± 0.01
G3	15.5 ± 0.17	6.56 ± 0.13	5.64 ± 0.07	11.9 ± 0.11
G4	7.59 ± 0.51	5.15 ± 0.10	5.34 ± 0.25	3.61 ± 0.11
Isomaltose	0.003 ± 0.0005	0.008 ± 0.0005	0.056 ± 0.003	0.002 ± 0.0004
Nigerose	0.015 ± 0.003	0.037 ± 0.004	0.018 ± 0.006	0.011 ± 0.008
Kojibiose	0.007 ± 0.001	0.039 ± 0.003	0.004 ± 0.001	0.011 ± 0.0004
Trehalose	0.004 ± 0.002	0.009 ± 0.003	ND	0.005 ± 0.001
pNPGlc	2.61 ± 0.11	4.42 ± 0.09	2.98 ± 0.03	0.48 ± 0.013

Reaction rate for hydrolysis is shown using 2 mM substrate concentrations. G2, G3, G4, pNPGlc represent maltose, maltotriose, maltotetraose, and *p*-nitrophenyl α-glucopyranoside, respectively. ND = too small to quantify. Values presented as average ± SD from three independent experiments.

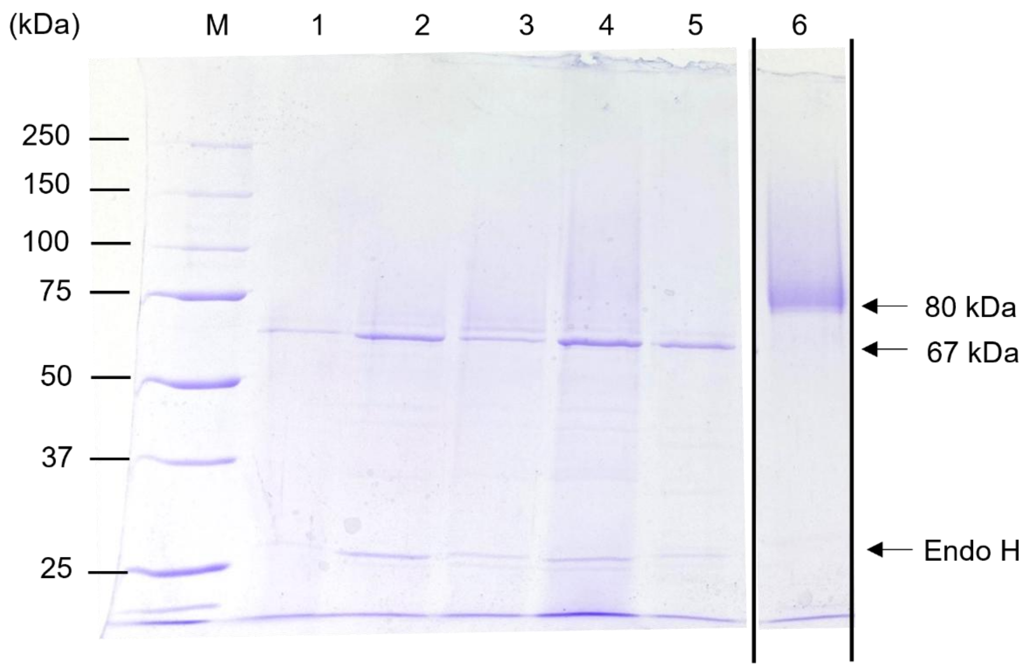

Figure S1. SDS-PAGE analysis of purified AaMalI and its mutants. Purified samples with endo H treated were analyzed. The protein was stained with CBB. Lane M, protein size marker. Lane 1, wild type. Lane 2, Y223H. Lane 3, P222N. lane 4, A407E; lane 5, P222N/Y223H. Lane 6 is purified AaMalI (2 μg) without endo-H treated.

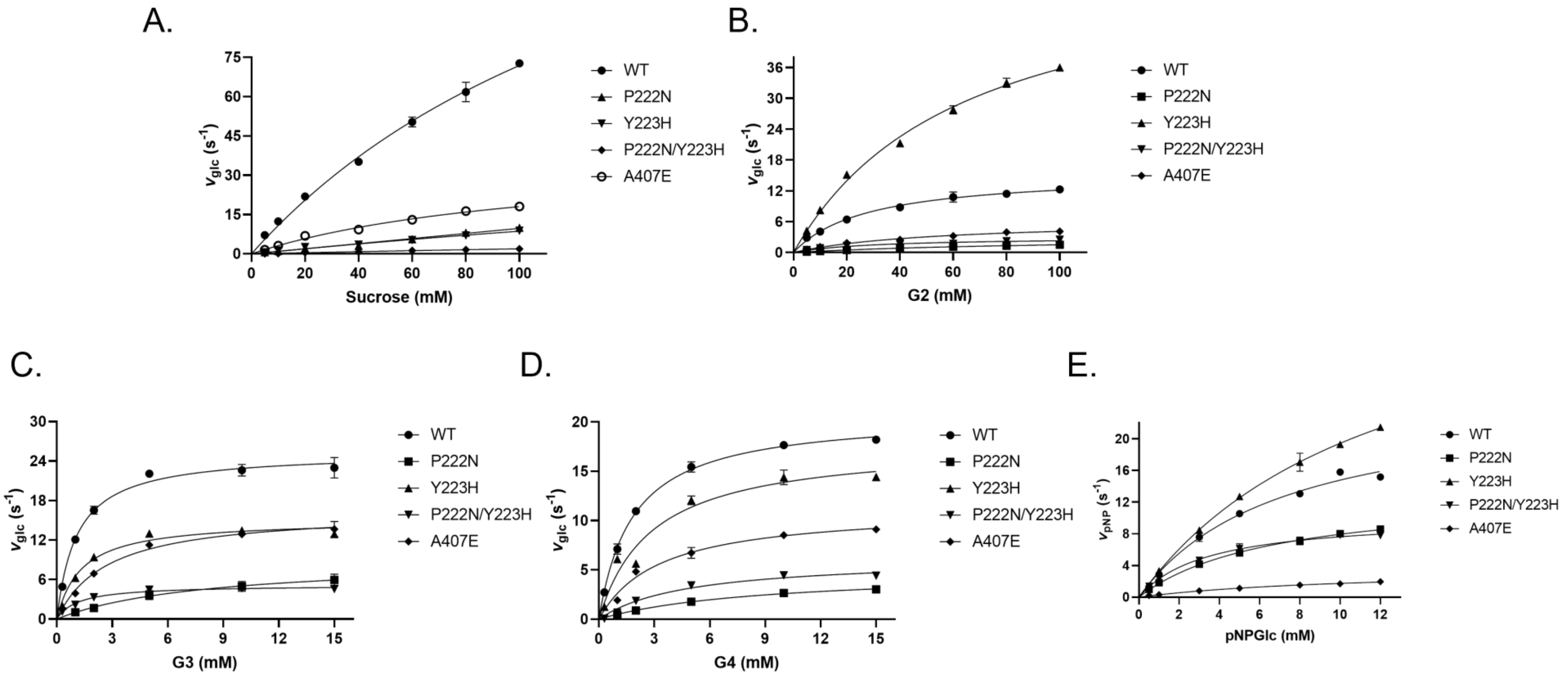

Figure S2. Kinetic analysis of the reaction of AaMalI wild-type (WT) and mutant enzymes (P222N, Y223H, P222N/Y223H, and A407E) with sucrose, G2, G3, G4 and pNPGlc. Each data point represents the mean ± SD (n = 3). Curves were fitted to the Michaelis–Menten equation using GraphPad Prism. The *s*-*v* plots for the reactions with (A) sucrose, (B) G2, (C) G3, (D) G4 and (E) pNPGlc are shown. D-glucose releasing velocity (v_{glc}) are presented, and for pNPGlc, pNP releasing velocity (v_{pNP}) is indicated.

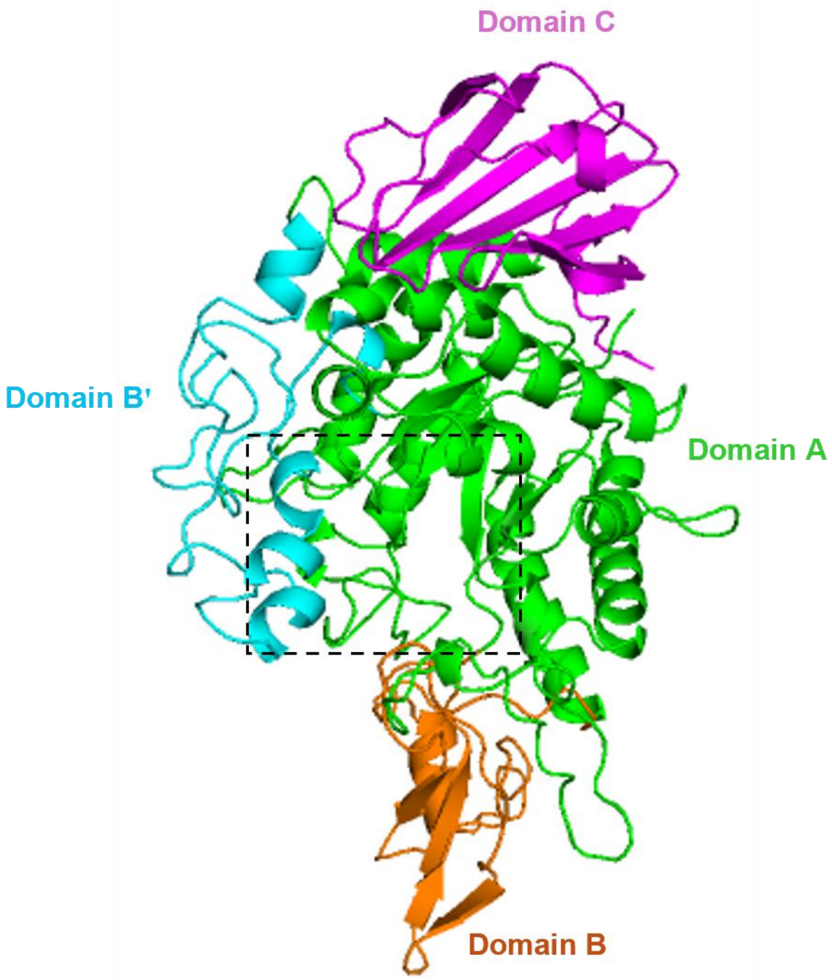

Figure S3. Overall structure of AaMalI. The structure of AaMalI was predicted using AlphaFold3, showing four distinct domains. Domains A, B, B', and C are colored in green, orange, cyan, and magenta, respectively. The catalytic pocket is located in the groove adjacent to domain A and is indicted by the dashed box.

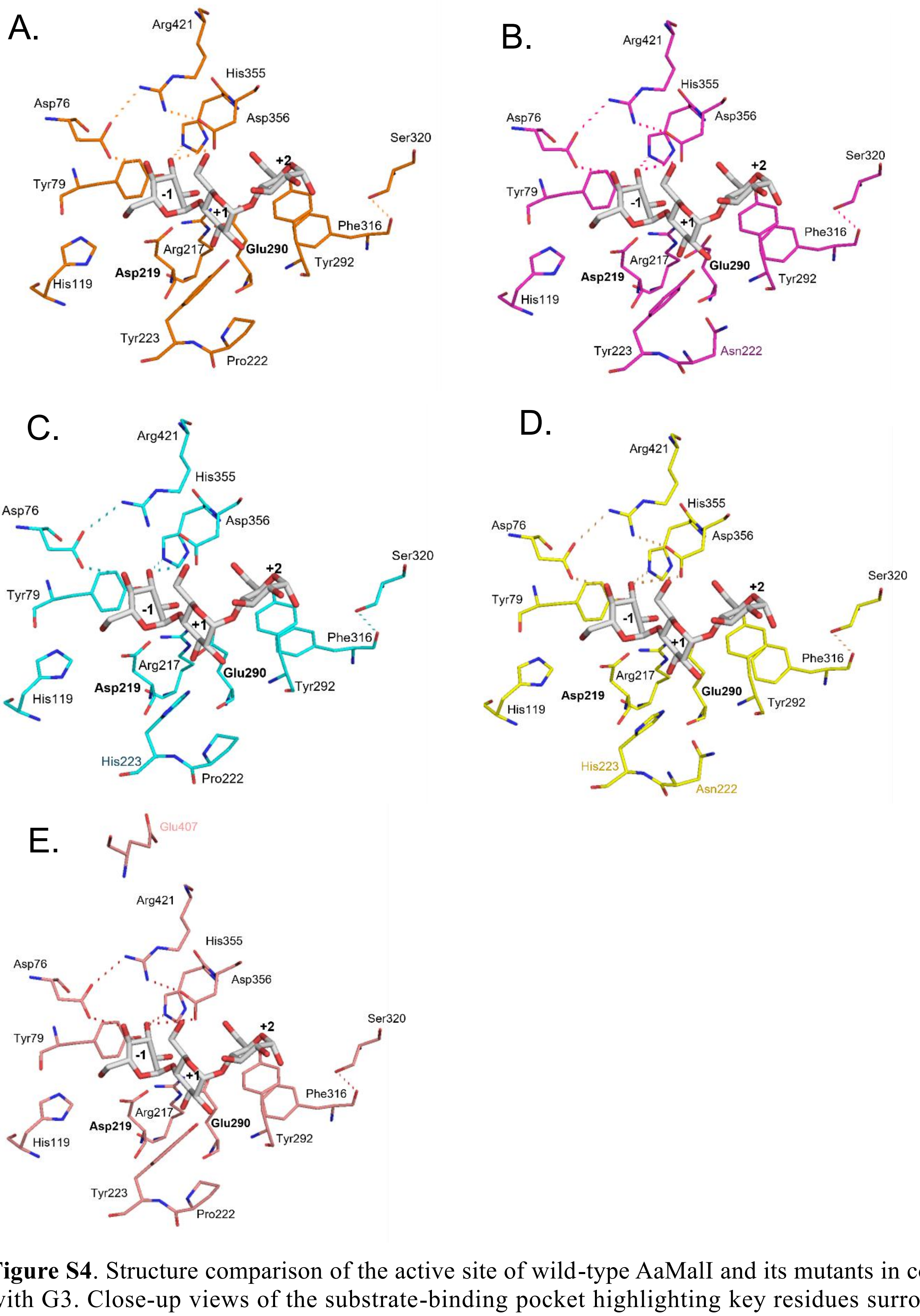

Figure S4. Structure comparison of the active site of wild-type AaMalI and its mutants in complex with G3. Close-up views of the substrate-binding pocket highlighting key residues surrounding subsites -1, +1 and +2 are shown. Wild type (A), P222N (B), Y223H (C), P222N/Y223H (D), and A407E (E) are presented. Conserved residues and mutated residues are shown in stick representation. Asp219 and Glu290 act as nucleophile and catalytic acid/base, respectively. Hydrogen-bond interactions are indicated by dotted lines. G3 derived from PDB entry 5ZCD is shown as gray sticks.

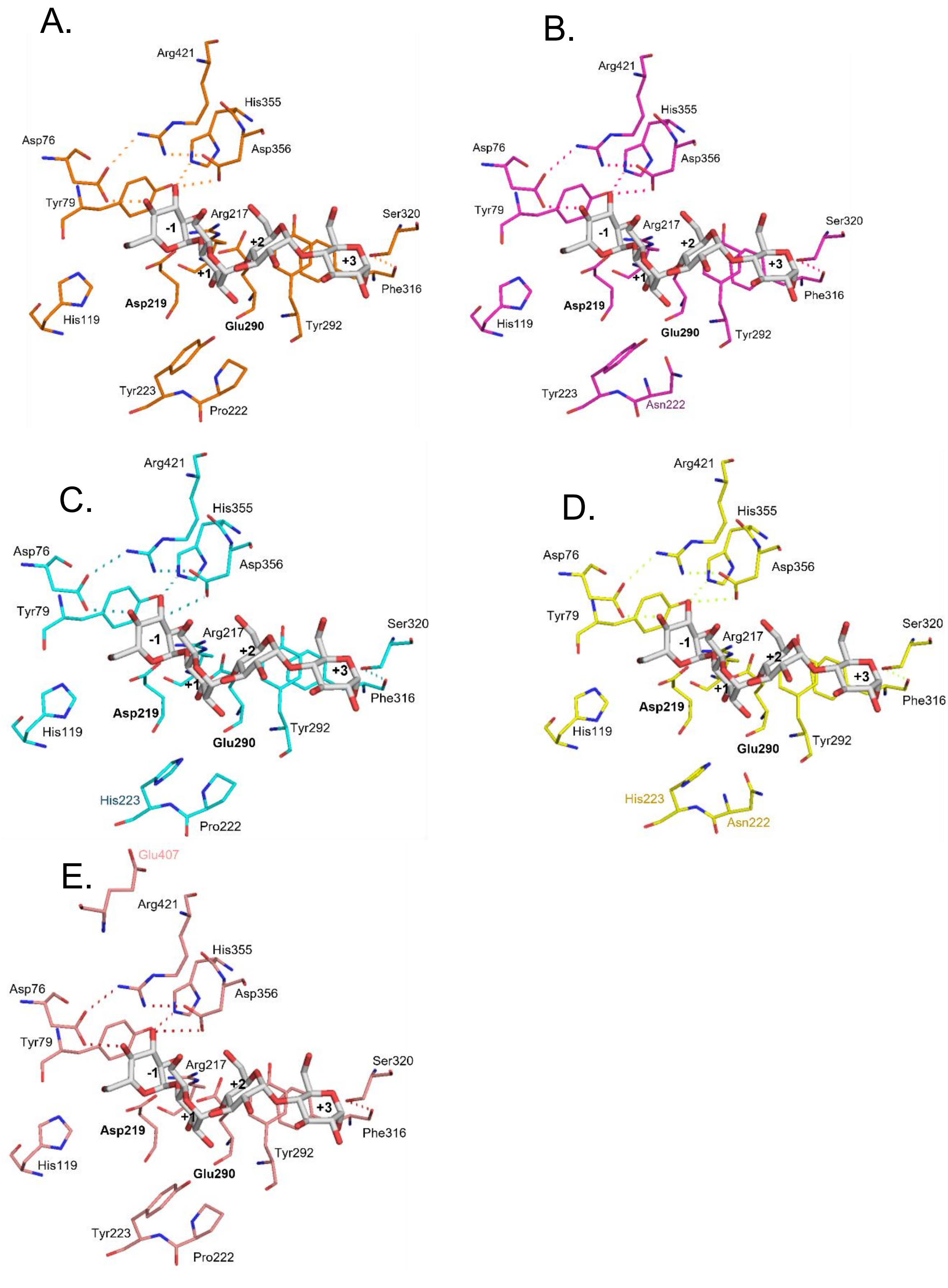

Figure S5. Structure comparison of the active site of wild-type AaMalI and its mutants in complex with G4. Close-up views of the substrate-binding pocket highlighting key residues surrounding subsites -1, +1, +2 and +3 are shown. Wild type (A), P222N (B), Y223H (C), P222N/Y223H (D), and A407E (E) are presented. Conserved residues and mutated residues are shown in stick representation. Asp219 and Glu290 act as nucleophile and catalytic acid/base, respectively. Hydrogen-bond interactions are indicated by dotted lines. G4 derived from PDB entry 5ZCE is shown as gray sticks.
